# Searching for molecular hypoxia sensors among oxygen-dependent enzymes

**DOI:** 10.7554/eLife.87705

**Published:** 2023-07-26

**Authors:** Li Li, Susan Shen, Philip Bickler, Matthew P Jacobson, Lani F Wu, Steven J Altschuler

**Affiliations:** 1 Department of Pharmaceutical Chemistry, University of California San Francisco, San Francisco San Francisco United States; 2 Department of Psychiatry, University of California, San Francisco San Francisco United States; 3 Hypoxia Research Laboratory, University of California San Francisco, San Francisco San Francisco United States; 4 Center for Health Equity in Surgery and Anesthesia, University of California San Francisco, San Francisco San Francisco United States; 5 Anesthesia and Perioperative Care, University of California San Francisco, San Francisco San Francisco United States; University of Michigan United States; Iowa State University United States

**Keywords:** hypoxia sensors, oxygen, oxygen-dependent enzymes, hypoxia

## Abstract

The ability to sense and respond to changes in cellular oxygen levels is critical for aerobic organisms and requires a molecular oxygen sensor. The prototypical sensor is the oxygen-dependent enzyme PHD: hypoxia inhibits its ability to hydroxylate the transcription factor HIF, causing HIF to accumulate and trigger the classic HIF-dependent hypoxia response. A small handful of other oxygen sensors are known, all of which are oxygen-dependent enzymes. However, hundreds of oxygen-dependent enzymes exist among aerobic organisms, raising the possibility that additional sensors remain to be discovered. This review summarizes known and potential hypoxia sensors among human O_2_-dependent enzymes and highlights their possible roles in hypoxia-related adaptation and diseases.

## Introduction

In aerobic organisms, the dioxygen molecule (O_2_) is essential for many biochemical pathways, particularly as the final electron acceptor for bioenergetics. Hypoxia—conditions of decreased O_2_ availability—is both an essential stimulus for normal development and a pathological trigger of cellular dysfunction and eventual cell death for humans and other mammals (Bickler and Buck, 2007). To maintain O_2_ homeostasis, aerobic organisms have developed diverse cellular mechanisms for sensing and responding to alterations in O_2_ level. For multiorgan organisms, the term ‘hypoxia’ is often loosely used to describe decreased O_2_ levels. More precisely, the term ‘tissue hypoxia’ is meaningful when used in comparison to the baseline for the tissue. Physiological tissue O_2_ (physoxia, the typical range of function), physiological hypoxia (reduction or fluctuation of pO_2_ into a range at which adaptation is possible), and hypoxia with pathological impact (pO_2_ at which cellular injury and death occur) are often cited as ~5, 2, and 1%, respectively, for humans (McKeown, 2014). However, these values can vary widely across tissues and even within a tissue and are affected by tissue-level regulation (e.g., blood flow) and cellular effects (e.g., changes in metabolic state) (Table 1; McKeown, 2014; Ortiz-Prado et al., 2019; Carreau et al., 2011; Jagannathan et al., 2016; Cigognini et al., 2016; Donovan et al., 2010; Mas-Bargues et al., 2019). Here, we focus on O_2_ sensing in humans, using the term ‘hypoxia’ to denote decreased O_2_ level relative to physoxia, that is, encompassing both physiological hypoxia and hypoxia with pathological impact.

**Table 1.** Physiological O_2_ distribution in different organs/tissues*.

Organ/tissue	%O_2_	pO_2_ (mmHg)	Concentration(μM)
Ambient air	21	160	206
Alveoli	14	104	134
Arterial blood	13	100	129
Kidney	4–9.5	30–73	39–94
Liver	4–7	30–54	39–69
Heart	2–6	15–46	19–59
Brain	3–5	23–39	29–50
Small intestine	2–9	15–69	19–89
Large intestine	0–6	0–46	0–59
Bone marrow	1.5–7	11–54	14–69

* The O_2_ levels in different organs are adjusted from references Burmester and Hankeln, 2014; Lecomte et al., 2005; Hatefi, 1985; Zaccara et al., 2019; Ball et al., 2014 and the partial pressure and concentration are calculated according to references Ortiz-Prado et al., 2019; Carreau et al., 2011; Jagannathan et al., 2016; Cigognini et al., 2016; Donovan et al., 2010; Mas-Bargues et al., 2019; Place et al., 2017.

Discovery of the PHD-HIF-pVHL pathway was pivotal to understanding hypoxia responses and has been reviewed extensively (Majmundar et al., 2010; Kaelin and Ratcliffe, 2008; Ivan and Kaelin, 2017; Schofield and Ratcliffe, 2004). Briefly, in normoxia, prolyl hydroxylase domain proteins (PHDs) use O_2_ as a substrate to hydroxylate prolines on the transcription factor hypoxia-inducible factor α subunit (HIFα, i.e., HIF1α or HIF2α). The hydroxylated form of HIFα is recognized by the E3 ubiquitin ligase pVHL (von Hippel-Lindau protein), which promotes degradation of HIFα. By contrast, in hypoxia, the decreased catalytic activity of PHDs results in decreased hydroxylation and hence decreased pVHL recognition of HIFα, promoting the accumulation of HIFα. HIFα then translocates to the nucleus and, as a heterodimer with HIF1β, regulates transcription of a broad range of target genes. Thus, PHDs directly sense a decrease in the availability of molecular O_2_ and transduce this signal to downstream effectors.

What defines a molecular hypoxia sensor? In engineering, a sensor is a device that detects changes to a physical property and transmits this information so that a system can respond to this change. Here, by analogy to human-engineered sensors, we define biological hypoxia sensors as proteins that (1) directly interact with O_2_ molecules, (2) have activities that are strongly affected by physiological hypoxia, and (3) are coupled to downstream responses that depend on changes of their activities. Of note, many proteins respond to hypoxia by acting downstream of a sensor (e.g., HIF acting downstream of PHD) or by responding to changes in the cellular redox state. In this review, we specifically exclude these as they are not direct sensors of molecular O_2_ —that is, their response to changes in O_2_ levels does not involve direct interaction with O_2_ (Bickler and Donohoe, 2002).

Strong candidates for hypoxia sensors include O_2_-dependent enzymes, which by definition meet criterion 1. These enzymes constitute a mechanistically, structurally, and biologically diverse group of proteins. There are a number of reviews on the enzymology (Islam et al., 2018; Palfey and McDonald, 2010; Decker and Solomon, 2005; Jasniewski and Que, 2018; Biringer, 2020; Ferguson-Miller and Babcock, 1996; Finney et al., 2014; Bassan et al., 2003; Guengerich, 2007; Ponnaluri et al., 2013; Ivanov et al., 2010; Daff, 2010; Wikström et al., 2018; Huang and Groves, 2018; Romero et al., 2018; Sono et al., 1996; Martinez and Hausinger, 2015; Roberts and Fitzpatrick, 2013; Itoh, 2006; Solomon et al., 2001; Bugg, 2001; Abu-Omar et al., 2005), biological function (Schofield and Ratcliffe, 2004; Islam et al., 2018; Paton and Ntambi, 2009; Shmakova et al., 2014; Danielson, 2002; Donkó et al., 2005; Bundred et al., 2018; Fletcher and Coleman, 2020; Kuhn et al., 2015; Zhuang et al., 2015; Kooistra and Helin, 2012; Mashima and Okuyama, 2015; Wu and Zhang, 2017; Johansson et al., 2014; Daubner et al., 2011; Fong and Takeda, 2008; Markolovic et al., 2015), and evolution (Danielson, 2002; Taylor and McElwain, 2010; Chandrasekharan and Simmons, 2004; Wilks, 2002) of individual subclasses of O_2_-dependent enzymes. Here, we provide a global map of human O_2_-dependent enzymes in potential hypoxia sensing. We first survey the broad categories and then discuss specific members that are known or speculated hypoxia sensors. Finally, we investigate the links between O_2_-dependent enzymes and hypoxia-related evolutionary adaptations and diseases.

### O_2_-dependent enzymes as hypoxia sensor candidates

We start by providing background and taxonomies for considering the three basic ‘sensor’ requirements discussed above.

**Figure 1.**
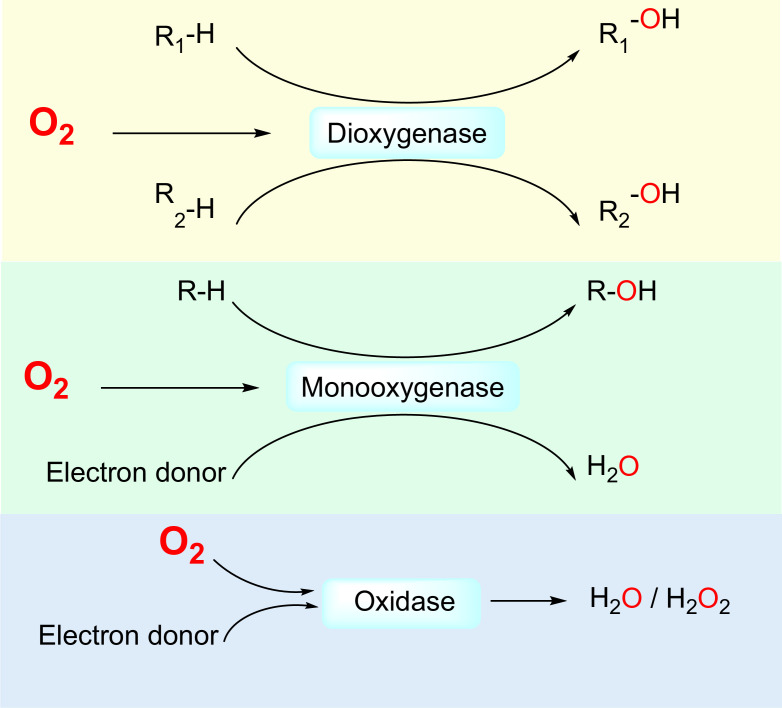
Three classes of by O_2_-dependent enzymes (dioxygenases, monooxygenases, and oxidases) and the reactions they catalyze. Dioxygenases catalyze the insertion of both oxygen atoms of the dioxygen molecule into substrates. Monooxygenases catalyze the insertion of one oxygen atom of the dioxygen molecule into a substrate and the other oxygen atom is reduced to H_2_O. Oxidases catalyze the reduction of dioxygen to H_2_O or H_2_O_2_.

First, O_2_-dependent enzymes directly interact with O_2_ molecules as one of the substrates. Non-enzymatic proteins that directly interact with O_2_, for example, globins, have been reviewed elsewhere (Burmester and Hankeln, 2014; Lecomte et al., 2005). In humans, 221 enzymes are known or likely to be O_2_-dependent (Supplementary file 1), that is, utilizing O_2_ as an electron acceptor for the oxidation of other substrates. Based on their catalyzed reactions, O_2_-dependent enzymes can be divided into three subclasses: *dioxygenases*, which catalyze the insertion of both oxygen atoms of the O_2_ molecule into substrates; *monooxygenases*, which catalyze the insertion of one oxygen atom of the O_2_ molecule into a substrate and the reduction of the other oxygen atom to H_2_O; and *oxidases*, which catalyze the reduction of O_2_ molecules to 2H_2_O or H_2_O_2_ (Figure 1).

Second, O_2_-dependent enzymes have diverse mechanisms for utilizing O_2_ as a substrate, resulting in different sensitivities to O_2_ concentrations. Sensitivity is determined, in part, by the binding affinity of O_2_ with the enzyme’s catalytic center. Most O_2_-dependent enzymes (177/221) use, or are speculated to use, O_2_-binding metal ions at their catalytic centers. Factors affecting the O_2_-binding affinity include the metal center (iron or copper in humans), ligands (enzyme residues and other substrates) for the metal center, and the environment of the catalytic pocket. The other O_2_-dependent enzymes with known non-metal catalytic centers (37/221) utilize flavin adenine dinucleotide (FAD) or flavin mononucleotide (FMN) to activate O_2_. For these enzymes, the accessibility of O_2_ to FAD or FMN at the catalytic center affects the binding affinity. Dioxygenases, monooxygenases, and oxidases can be further subdivided by their catalytic centers (Table 2). Ultimately, these mechanisms affect the threshold at which enzymatic activities are saturated with O_2_, thus determining whether the enzyme’s activities are strongly affected by physiological-range hypoxia.

**Table 2.** Categories of O_2_-dependent enzymes.

Category	Subcategory by catalytic center	Metal species at catalytic center	Ligands for the metal species at catalytic center (cofactor/substrate and enzyme residues)	Number of enzymes
Dioxygenase	2-OG-dependent dioxygenase	Fe	2-OG, His, His, Asp/Glu	59
	Heme-dependent dioxygenase	Fe	Heme, His	5
	Lipoxygenase	Fe	His, His, His, Ile, His/Asa/Asn/none	6
	Others	Fe	His, His, His/Asp/Glu*	10
Monooxygenase	Heme-dependent monooxygenase	Fe	Heme, Cys/His/Glu	61
	Non-Heme Fe-dependent monooxygenase	Fe	His, His, His/Asp/Glu*	9
	Cu-dependent monooxygenase	Cu	His, His, Met	5
	Flavin-dependent monooxygenase	None (uses flavin)	N/A	12
	Others†	N/A	N/A	2
Oxidase	Heme-copper	Fe and Cu	His, His, His for Cu; Heme and His for Fe	1
	Fe-dependent oxidase	Fe	Varies	14
	Cu-dependent oxidase	Cu	Varies	7
	Flavin-dependent oxidase	None (uses flavin)	N/A	25
	Others†	N/A	N/A	5

* Substrates/cofactor ligands for this category varies for each member depending on the reaction it catalyzes.

† Members in this category are not fully studied.

Third, O_2_-dependent enzymes regulate diverse cellular processes: (1) oxidative phosphorylation is responsible for mitochondrial ATP production and cellular survival (Hatefi, 1985); (2) post-translational modifications (hydroxylation, demethylation, or thiol oxidation) of proteins can regulate protein conformation, stability, and activity (Schofield and Ratcliffe, 2004; Bundred et al., 2018; Fletcher and Coleman, 2020; Kooistra and Helin, 2012; Johansson et al., 2014); (3) hydroxylation and demethylation of DNA/RNA can regulate DNA damage repair, epigenetic modifications, and transcription/translation (Wu and Zhang, 2017; Zaccara et al., 2019); (4) metabolism of amino acids and lipids can maintain cellular hemostasis and regulate cellular pathways through signaling molecules (Paton and Ntambi, 2009; Kuhn et al., 2015; Mashima and Okuyama, 2015; Daubner et al., 2011; Chandrasekharan and Simmons, 2004; Ball et al., 2014); and (5) metabolism of xenobiotics can regulate drug clearance and detoxification (Danielson, 2002; Poulos and Johnson, 2005). Typically, dioxygenases have macromolecules as substrates and regulate cellular processes at a transcriptional or translational level (Schofield and Ratcliffe, 2004; Islam et al., 2018; Bundred et al., 2018; Fletcher and Coleman, 2020; Kooistra and Helin, 2012; Wu and Zhang, 2017; Johansson et al., 2014; Hancock et al., 2015), while monooxygenases and oxidases often have small molecules as substrates and function in metabolism (Romero et al., 2018; Paton and Ntambi, 2009; Danielson, 2002; Daubner et al., 2011). Together, O_2_-dependent enzymes are integral to a plethora of physiological processes in aerobic animals.

Candidate hypoxia sensors can be identified among the O_2_-dependent enzymes, in part by the binding affinity between O_2_ and the enzyme as quantified by the O_2_ K_m_ value, which suggests the level at which the enzyme is most sensitive to changes in O_2_ (Kaelin and Ratcliffe, 2008; Schofield and Ratcliffe, 2004; Shmakova et al., 2014; Hancock et al., 2015; Wilson et al., 2020; Holdsworth and Gibbs, 2020; Baik and Jain, 2020). (The Km value, also known as the Michaelis constant, is the concentration of a substrate at which an enzymatic reaction rate is 50% of the maximum reaction rate. A larger K_m_ value reflects lower O_2_ affinity.) Importantly, the measured K_m_ value is affected by the measurement method, for example, mass spectrometry vs. isotope assays. Besides the Km value, other cellular factors such as the concentration and conformation of the enzyme, as well as concentrations of other substrates or products, also affect the net enzymatic activity and hence the downstream effects of the enzyme. Beyond cellular-level effects, whether an O_2_-dependent enzyme functions as a hypoxia sensor in vivo can depend on the tissue pO_2_ context (Table 1). Taken together, whether an O_2_-dependent enzyme functions as a hypoxia sensor in vivo depends not only on the O_2_ K_m_ value but also on multiple other factors.

### O_2_-dependent enzymes that are known or potential hypoxia sensors

**Figure 2.**
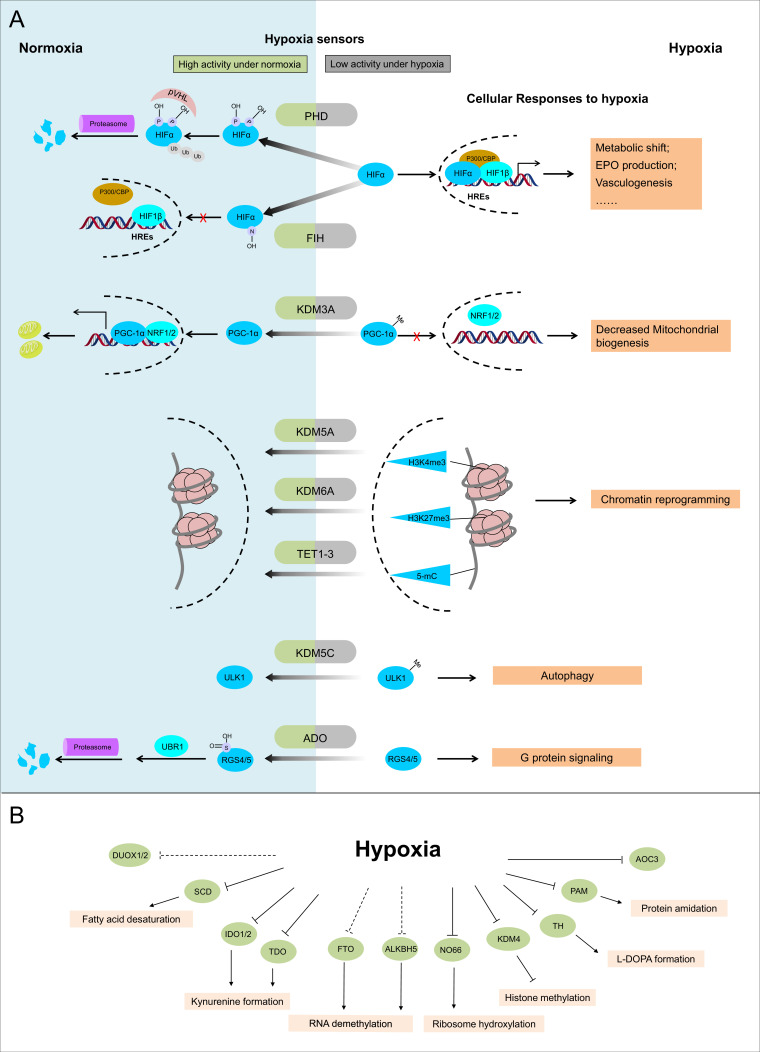
Known and candidate sensors for hypoxia inside O_2_-dependent enzymes. (**A**) Known hypoxia sensors and their corresponding cellular responses to hypoxia. Decreased O_2_ concentration inhibits activities of hypoxia sensors in O_2_-dependent enzyme category and results in changes downstream signaling pathway as the cellular response to hypoxia. PHD catalyzes the hydroxylation at two prolyl residues of HIFα, and then the hydroxylated HIFα is recognized and ubiquitylated by pVHL. Following ubiquitilation, HIFα is degraded by proteasome. During hypoxia, activity of PHD is diminished and HIFα is stabilized. Accumulated HIFα translocates to the nucleus, and in dimerization with HIF1β, recruits other transcriptional coactivators (p300, CBP), binds with the hypoxia response elements (HREs) and activates the transcription of HIF target genes. The products of these genes participate in adaptation to hypoxia including metabolic shift, EPO production, vasculogenesis, etc. FIH catalyzes the asparaginyl hydroxylation of HIFα, and this hydroxylation inhibits HIFα from recruiting transcriptional coactivators. Compared with PHD, FIH is inhibited by more severe hypoxia. KDM3A catalyzes the demethylation of K244 monomethylation of PGC-1α, which is a transcriptional coactivator and regulates mitochondrial biogenesis. Under normoxia, PGC-1α binds with transcriptional factor NRF1/2 and activates the transcription of nucleus-encoded mitochondrial genes. Under hypoxia, the inhibited activity of KDM3A leads to accumulation of K224 monomethylation at PGC-1α. The maintained monomethylation at K224 of PGC-1α reduces its binding ability with NRF1/2 and results in decreased mitochondrial biogenesis. KDM5A catalyzes the demethylation at Lys4 of histone H3 (H3K4). Hypoxia inhibits its activity and results in the hypermethylation at H3K4, which is responsible for the gene activation. Similarly, hypoxia also inhibits KDM6A, and results in the hypermethylation at its target site H3K27 and gene repression. TET methylcytosine dioxygenases (TET1, TET2, and TET3) catalyze conversion of DNA 5-methylcytosine (5-mC) to the 5-hydroxymethylcytosine (5hmC) and mediates DNA demethylation. Hypoxia reduces TET activity and causes DNA hypermethylation. Together, these proteins sense hypoxia and lead to transcription alteration by chromatin reprogramming. KDM5C catalyzes the demethylation of ULK1 R170me2s, which regulates ULK1 activity. Under normoxia, R170me2s of ULK1 is removed by KDM5C and ULK1 remains inactive. Under hypoxia, the inhibited activity of KDM5C leads to accumulation of ULK1 R170me2s, and results in ULK1 activation and autophagy induction. ADO catalyzes the thiol oxidation at the N terminal Cys of a protein, which then triggers its degradation through N-degron pathway. Hypoxia inhibits the activity of ADO and leads to the stabilization of its substrates. One of the identified ADO substrates is RSG4/5, regulators of the G protein signaling. Stabilization of RGS4/5 results in the modulation of G-protein-coupled calcium ion signaling. (**B**) Candidate O_2_ sensors with reduced enzymatic activities in hypoxia. Hypoxia leads to: inhibition of KDM4A and KDM4B and accumulated hypermethylation at H3K9; inhibition of SCD and increased cellular fatty acid saturation; inhibition of IDO and changes of immunoregulation; inhibition of PAM and reduced protein amidation; in vitro inhibition of RIOX1 and RIOX2 which are responsible for ribosome hydroxylation; in vitro inhibition of AOC3; RNA hypermethylation possibly through inhibition of FTO/ALKBH5; potential inhibition of DUOX1 and DUOX2. PHD: prolyl hydroxylase domain-containing protein; HIF: hypoxia-inducible factor; pVHL: von Hippel-Lindau protein E3 ligase; CBP, cyclic-AMP response element binding protein binding protein; EPO: erythropoietin; FIH: factor inhibiting HIF1; KDM: JmjC (Jumonji C) domain lysine demethylase; PGC: peroxisome proliferator-activated receptor gamma coactivator; NRF: nuclear respiratory factor; TET: ten-eleven translocation methylcytosine dioxygenases; ADO: cysteamine (2-aminoethanethiol) dioxygenase; RGS: regulators of G protein signalling; SCD: stearoyl-CoA desaturases; IDO: indoleamine 2,3-dioxygenase; AOC: amine oxidase, copper containing; PAM: peptidylglycine α-amidating monooxygenase; RIOX: ribosomal oxygenase, FTO: fat mass and obesity-associated protein; ALKBH: AlkB homolog; DUOX: dual oxidase.

Below, we classify dioxygenases, monooxygenases, and oxidases into different subgroups based on their catalytic centers and discuss known (Figure 2A) and potential (Figure 2B) hypoxia sensors in each subgroup.

### Dioxygenases

Based on their catalytic centers, the dioxygenase family members can be further classified into 2-OG-dependent dioxygenase, heme-dependent dioxygenases, lipoxygenases, and other dioxygenases (Table 2).

#### 2-Oxyglutarate (2-OG)-dependent dioxygenases

**Figure 3.**
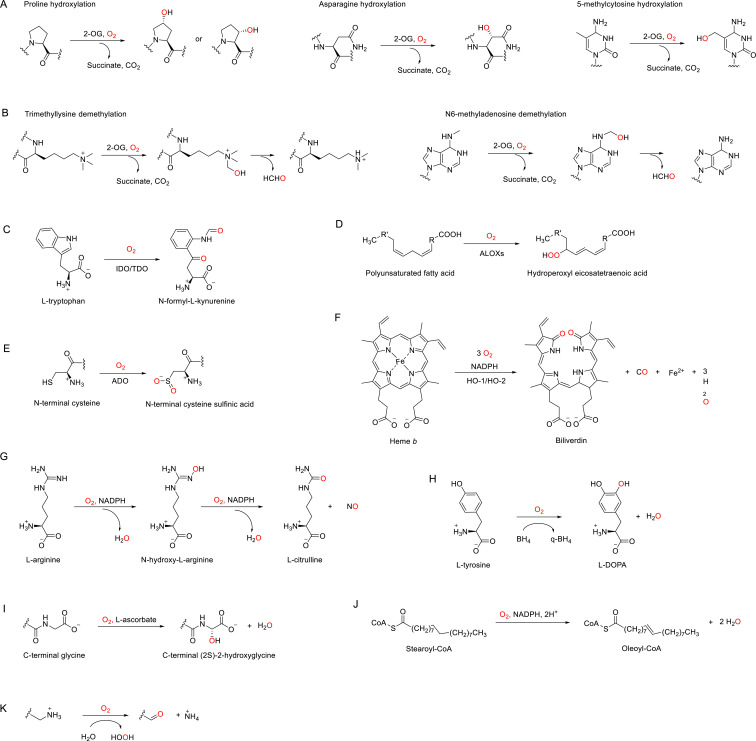
Enzymatic reactions catalyzed by discussed O_2_-dependent enzymes. (**A**) Examples of hydroxylation reactions catalyzed by 2-OG-dependent dioxygenases. (**B**) Examples of demethylation reactions catalyzed by 2-OG-dependent dioxygenases. (**C–K**) Reactions catalyzed by indoleamine 2,3-dioxygenase (IDO)/tryptophan 2,3-dioxygenase (TDO) (**C**), arachidonate lipoxygenases (ALOXs) (**D**), (2-aminoethanethiol) dioxygenase (ADO) (**E**), heme oxygenases (HOs) (**F**), nitric oxide synthases (NOSs) (**G**), tyrosine 3-hydroxylase (TH) (**H**), peptidylglycine α-amidating monooxygenase (PAM) (**I**), stearoyl-CoA desaturase 1 (SCD1), (**J**) and copper amine oxidases (CAOs) (**K**).

In humans, there are ~60 identified or postulated dioxygenases (Table 2, Supplementary file 1) that use 2-OG as the co-substrate to catalyze the hydroxylation of their primary substrates, which include proteins, nucleic acids, and lipids (Figure 3A, Figure 3—figure supplement 1; Islam et al., 2018; Fletcher and Coleman, 2020; Rose et al., 2011). We note that when hydroxylation occurs on the carbon of an N-methyl group, this can lead to demethylation, which occurs through spontaneous fragmentation to formaldehyde and the demethylated product (Figure 3B; Islam et al., 2018; Fletcher and Coleman, 2020; Rose et al., 2011).

**Figure 3—figure supplement 1.**
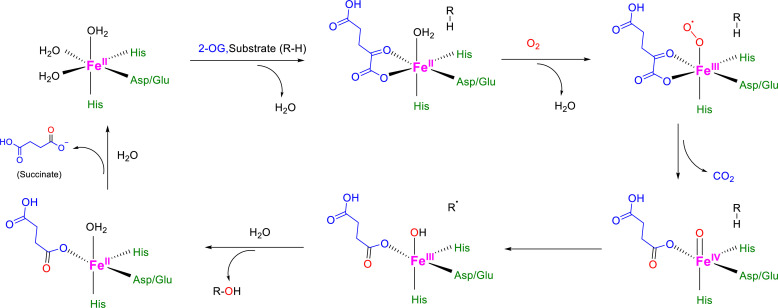
Catalytic mechanism for 2-OG-dependent dioxygenases. 2-OG-dependent dioxygenases share consensus mechanisms for the catalyzed hydroxylation (Islam et al., 2018; Fletcher and Coleman, 2020; Rose et al., 2011): the Fe(II) at the catalytic center is initially coordinated by 2 His side chains and a carboxylate from Glu or Asp, plus three additional H_2_O molecules to complete the octahedral coordination geometry. Then, bidentate coordination of 2-OG to Fe(II) replaces 2 H_2_O molecules, and the third Fe(II)-bound H_2_O molecule leaves after the binding of the primary substrate into the active site, making a vacant coordination site for O_2_. After the binding and activation of O_2_ at the Fe(II) center, one of the O_2_ atoms inserts into the primary substrate for hydroxylation, while the other O_2_ atom facilitates the oxidative decarboxylation of 2-OG, forming succinate and CO_2_ as co-products.

**Figure 3—figure supplement 2.**
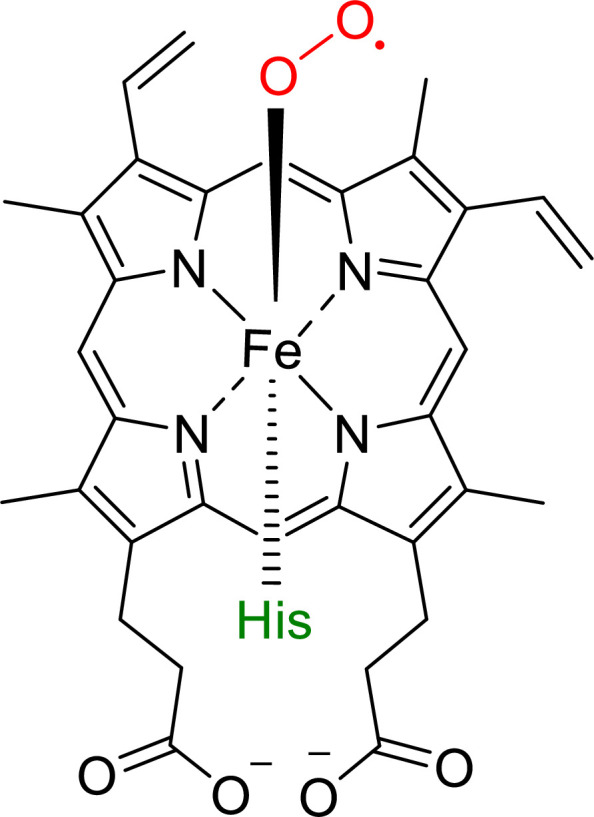
O_2_-binding sites for dioxygenases using heme. The heme Fe(II) is coordinated by the four N atoms from the porphyrin and one N atom from one histidyl residue in the catalytic pocket, leaving the vacant coordination site for O_2_ binding and activation (Singleton et al., 2014).

**Figure 3—figure supplement 3.**
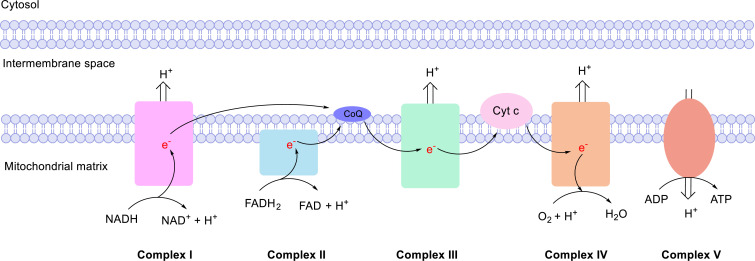
Mitochondrial electron transport chain (ETC). The ETC consists of NADH ubiquinone oxireductase (Complex I), succinate dehydrogenase (Complex II), CoQH_2_-cytochrome c reductase (Complex III), and cytochrome *c* oxidase (Complex IV). In the ETC, electrons are transported from the NADH or FADH_2_ to ubiquinone at Complex I or II, then to cytochrome *c* at Complex III, and finally to O_2_ at Complex IV. This process is coupled with ATP generation at ATP synthase (Complex V) to form the oxidative phosphorylation (OxPhos) process, which is the major source of energy production.

Among O_2_-dependent enzymes, 2-OG-dependent dioxygenases are relatively well studied. A majority of members in this subgroup catalyze hydroxylation or demethylation on proteins, DNA, and RNA, and are involved in the regulation of transcription and translation (Islam et al., 2018; Fletcher and Coleman, 2020; Rose et al., 2011). We focus on three subgroups relevant to hypoxia biology: direct HIF modulators, epigenetic modulators, and translational modulators. These subgroups encompass most known hypoxia sensors, including PHDs, factor inhibiting HIF (FIH1), lysine demethylases (KDMs), and ten-eleven translocation methylcytosine dioxygenases (TET1-3), as well as potential sensors that have impaired activities during hypoxia. For each subgroup, we highlight the most well-known sensors and propose additional, potential sensors.

#### Direct HIF modulators

These include PHDs (which catalyze prolyl hydroxylation of HIFα) and FIH (which catalyzes asparaginyl hydroxylation of HIFα) (Table 3).

**Table 3.** Direct HIF modulator in 2-OG-dependent dioxygenases.

Gene symbol	Protein name	Type of reaction	Hydroxylation sites in HIFα	Non-HIF substrate examples
EGLN1	PHD2	Prolyl hydroxylation	HIF1α Pro402, Pro564; HIF2α Pro405, Pro531; HIF3α Pro492	FLNA, Akt
EGLN2	PHD1	Prolyl hydroxylation	HIF1α Pro402, Pro564; HIF2α Pro405, Pro531; HIF3α Pro492	FOXO3, Cep192, TP53
EGLN3	PHD3	Prolyl hydroxylation	HIF1α Pro564; HIF2α Pro405, Pro531; HIF3α Pro492	ATF-4, ADRB2, TP53
HIF1AN	FIH1	Asparaginyl hydroxylation	HIF1α Asn803, HIF2α Asn847	IκBα, Notch, OTUB1, RIPK4

The PHD enzymes and their critical role in regulating the PHD–HIF-pVHL signaling pathway are a paradigm for cellular sensing and response to hypoxia (Figure 2A; Majmundar et al., 2010; Kaelin and Ratcliffe, 2008; Ivan and Kaelin, 2017; Schofield and Ratcliffe, 2004). In humans, HIF is composed of an α subunit (HIF1α, HIF2α, or HIF3α) and invariant β subunit (HIF1β), and there are three PHD isoforms, namely PHD1 (EGLN2), PHD2 (EGLN1), and PHD3 (EGLN3). These PHDs are canonical sensors that illustrate our criteria for O_2_ sensors.

First, PHDs directly interact with O_2_, utilizing O_2_ to hydroxylate prolines in the O_2_-dependent degradation domain (ODD) of HIFα (Epstein et al., 2001; Hirsilä et al., 2003).

Second, the enzymatic activities of PHDs are sensitive to cellular/tissue hypoxia. The O_2_-binding affinities of all three PHDs, represented by O_2_ Km values, have been measured in vitro with HIF1α peptides as substrates. Using a short 19-residue HIF1α fragment as the substrate, the reported O_2_ Km values for PHD1-3 are in the range of 229–1746 μM (Table 4; Hirsilä et al., 2003; Dao et al., 2009; Tarhonskaya et al., 2014). However, recent measurements using longer HIF1α fragments estimate O_2_ Km values for PHD2 in the range of 67–85 μM (Table 4; Ehrismann et al., 2007), corresponding to pO_2_ values in the (physoxia) range of 6–8% (Table 1), consistent with the sensitivities of PHDs to changes in physiological O_2_ concentrations.

**Table 4.** Reported Km values of O_2_-dependent enzymes. Km values vary based on the assay method and tested substrate. Some enzymes have multiple Km values listed, reflecting measurements from different studies.

Category	Enzyme*	Km for O_2_	Assay details	Reference
Dioxygenase	PHD2 (EGLN1)	**250** μM	In vitro radioactivity 2-OG turnover assay with HIF1α (556–574) peptide as substrate	Hirsilä et al., 2003
		**1746** μM	In vitro time-resolved fluorescence resonance energy transfer assay with P564-HIF1α peptide (DLEMLAPYIPMDDDFQL) as substrate	Dao et al., 2009
		**67** μM	In vitro O_2_ consumption assay with HIF1α (502–697) peptide as substrate	Ehrismann et al., 2007
		**81** μM	In vitro O_2_ consumption assay with HIF1α (530–698) peptide as substrate	Ehrismann et al., 2007
	**PHD1 (EGLN2**)	**230** μM	In vitro radioactivity 2-OG turnover assay with HIF1α (556–574) peptide as substrate	Hirsilä et al., 2003
	**PHD3**	**230** μM	In vitro radioactivity 2-OG turnover assay with HIF1α (556–574) peptide as substrate	Hirsilä et al., 2003
	KDM4E	197 μM	In vitro O_2_ consumption assay with ARK(me3)STGGK peptide as substrate	Cascella and Mirica, 2012
	KDM4A	173 μM	In vitro MALDI-TOF-MS assay with H31−15K9me3 peptide as substrate	Hancock et al., 2017
		57 μM	In vitro O_2_ consumption assay with ARK(me3)STGGK peptide substrate	Cascella and Mirica, 2012
		60 μM	In vitro radioactivity 2-OG turnover assay with histone H3(1–19)K9me3 as substrate	Chakraborty et al., 2019
	**KDM6A**	**180** μM	In vitro radioactivity 2-OG turnover assay with histone H3(21–44)K27(me3) as substrate	Chakraborty et al., 2019
	KDM4C	158 μM	In vitro O_2_ consumption assay with ARK(me3)STGGK peptide substrate	Cascella and Mirica, 2012
	KDM4B	150 μM	In vitro radioactivity 2-OG turnover assay with histone H3(1–19)K9me3 as substrate	Chakraborty et al., 2019
	**FIH**	**90** μM	In vitro radioactivity 2-OG turnover assay with HIF1α (788–822) peptide as substrate	Koivunen et al., 2004
	**KDM5A**	**90** μM	In vitro radioactivity 2-OG turnover assay with histone H3(1–21)K4me3 as substrate	Chakraborty et al., 2019
	**KDM3A**	**75 μM (7.6% O_2_**) †	In vitro demethylation-formaldehyde dehydrogenase-coupled reaction assay with K224-monomethylated PGC-1α peptide as substrate	Qian et al., 2019
	KDM5B	40 μM	In vitro radioactivity 2-OG turnover assay with histone H3(1–21)K4me3 as substrate	Chakraborty et al., 2019
	P4HA1	40 μM	Standard P4H activity assay with (Pro-Pro-Gly)_10_ (Peptide Institute) as a substrate	Hirsilä et al., 2003
	KDM5C	35 μM	In vitro radioactivity 2-OG turnover assay with histone H3(1–21)K4me3 as substrate	Chakraborty et al., 2019
	**TET1**	**30** μM	In vitro radioactivity 2-OG turnover assay with oligonucleotides containing a 5-mC as substrate	Laukka et al., 2016
		**3.0 μM (0.31% O_2_**) †	In vitro DNA hydroxymethylation assay with genomic DNA as substrate	Thienpont et al., 2016
	**TET2**	**30** μM	In vitro radioactivity 2-OG turnover assay with oligonucleotides containing a 5-mC as substrate	Laukka et al., 2016
		**5.2 μM (0.53% O_2_**) *	In vitro DNA hydroxymethylation assay with genomic DNA as substrate	Thienpont et al., 2016
	KDM5D	25 μM	In vitro radioactivity 2-OG turnover assay with histone H3(1–21)K4me3 as substrate	Chakraborty et al., 2019
	KDM6B	20 μM	In vitro radioactivity 2-OG turnover assay with histone H3(21–44)K27(me3) as substrate	Chakraborty et al., 2019
	IDO1	11.5–24 μM	In vitro O_2_ consumption assay with L-Trp as substrate	Kolawole et al., 2015
	PTGS1	10 μM (sheep)	In vitro radioactivity label assay with [1-^14^C]arachidonic acid as substrate	Juránek et al., 1999
	PTGS2	13 μM (mouse)	In vitro radioactivity label assay with [1-^14^C]arachidonic acid as substrate	Juránek et al., 1999
	ALOX5	13 μM (porcine)	In vitro radioactivity label assay with [1-^14^C]arachidonic acid as substrate	Juránek et al., 1999
	ALOX12	13 μM	In vitro radioactivity label assay with [1-^14^C]arachidonic acid as substrate	Juránek et al., 1999
	ALOX15	26 μM (porcrine)	In vitro radioactivity label assay with [1-^14^C]arachidonic acid as substrate	Juránek et al., 1999
	ALOX15	26 μM (rabbit)	In vitro radioactivity label assay with [1-^14^C]arachidonic acid as substrate	Juránek et al., 1999
	**ADO**	**>500** μM	In vitro UPLC-MS-TOF assay with RGS4(2–15) peptide as substrate	Masson et al., 2019
Monooxygenase	NOS1 (nNOS)	350 μM (rat)	In vitro heme-NO complex formation assay with L-arginine as substrate	Abu-Soud et al., 1996
	NOS2 (iNOS)	130 μM (mouse)	In vitro heme-NO complex formation assay with L-arginine as substrate	Abu-Soud et al., 2001
	NOS3 (eNOS)	4 μM (bovine)	In vitro heme-NO complex formation assay with L-arginine as substrate	Abu-Soud et al., 2000
		25 μM (bovine)	In vitro heme-NO complex formation assay with N-hydroxy-L-arginine as substrate	Abu-Soud et al., 2000
	TH	16.2 μM (low-activity state); 46.1 μM (high- activity state);	In vitro radioactivity label assay with ^3^H-tyrosine as substrate	Rostrup et al., 2008
		12.6–26.7 μM (low-activity state); 28.8–42.9 μM (high-activity state)^‡^;	In vitro oxygraphic assay with tyrosine as substrate	Rostrup et al., 2008
		2.6–3.9 μM (2–3 mmHg, rat) *	In vitro radioactivity label assay with ^3^H-tyrosine as substrate	Katz, 1980
	TPH1	3.9~12.9 μM (3–10 mmHg, rat) †	In vitro radioactivity label assay with ^3^H-tryptophan as substrate	Katz, 1980
	PAH	17 μM	In vitro oxygraphic assay with phenylalanine as substrate	Rostrup et al., 2008
	PAM	70 μM (rat)	In vitro radioactivity label assay with [α-^2^H2]-N-acylglycine of different chain length as substrates	McIntyre et al., 2010
Oxidase	Cytochrome *c* oxidase	<0.1 μM (rat)	In vitro O_2_ consumption assay measuring O_2_ consumption of purified rat mitochondria at low phosphate potential ([ATP]/[ADP]*[Pi])	Bienfait et al., 1975
		1–3 μM (rat)	In vitro O_2_ consumption assay measuring O_2_ consumption of purified rat mitochondria at high phosphate potential	Bienfait et al., 1975
		0.5 μM (mouse)	Cellular assay measuring the ‘apparent K (m)’ for O_2_ or p _50_ of respiration in 32D cells using high-resolution respirometry	Scandurra and Gnaiger, 2010
	AOC3	38 μM	In vitro enzymatic assay using purified human AOC3	Shen et al., 2012

* O_2_-dependent enzymes that are known sensors are highlighted in bold; that are reported to be inhibited under hypoxia are highlighted in a light orange background; that are reported to be associated with positive selections in high-altitude populations are highlighted in red (also see Table 6).

† Km of these enzyme were reported with units as % O_2_ or mmHg, and calculated according to Mas-Bargues et al., 2019; Place et al., 2017.

‡ Combined data for TH1/3/4 splicing isoforms.

Third, the decreased activity of PHDs during hypoxia triggers specific downstream responses (Figure 2A; Majmundar et al., 2010; Kaelin and Ratcliffe, 2008; Ivan and Kaelin, 2017; Schofield and Ratcliffe, 2004). Under normoxia, hydroxylated HIFα is recognized and polyubiquitinylated by the E3 ubiquitin ligase von Hippel-Lindau protein (pVHL), which then leads to proteasome-mediated degradation of HIFα. Under hypoxia, decreased O_2_ concentration suppresses the activity of PHDs. This allows HIFα to accumulate and translocate to nucleus, where it associates with the constitutively expressed HIF1β and forms the heterodimer transcriptional factor HIF. HIF then recruits transcriptional co-activators p300 and CREP-binding protein (CBP), binds with hypoxia-responsive elements (HREs) on DNA, and subsequently activates its target genes. Products of HIF-regulated genes are involved in multiple cellular and systematic adaptations to hypoxia, including metabolic shift from OXPHOS to glycolysis, redox homeostasis, angiogenesis, and erythropoiesis.

Although all PHD isoforms have similar O_2_ affinities, their different expression patterns and substrate preferences among the HIFα isoforms lead to differential regulation of hypoxia sensing and response by the PHD-HIF-pVHL pathway. Of the three PHD isoforms, PHD2 exerts the greatest control over the PHD-HIF-VHL pathway response to hypoxia (Berra et al., 2003) and it is ubiquitously expressed across mouse tissues and is the most abundant isoform (Appelhoff et al., 2004; Willam et al., 2006). PHD1 and PHD3 are more tissue-specific, with PHD1 most expressed in testis and PHD3 in heart (Willam et al., 2006). PHD2 favors HIF1α as substrate, while PHD1 and PHD3 favor HIF2α (Table 3; Appelhoff et al., 2004). Interestingly, PHD2 and PHD3 are themselves HIF target genes and can be induced during hypoxia to provide feedback regulation for the PHD-HIF-pVHL pathway (Epstein et al., 2001). The nonoverlapping and complex roles of different PHD isoforms are evidenced by knockout mice: Phd2-/- mice are embryonic lethal due to placental and heart defects, while postnatal whole-body knockout of Phd2 leads to polycythemia, increased angiogenesis, and heart defects (Takeda et al., 2006; Minamishima et al., 2008). Tissue-specific knockout of Phd2 in mouse heart or brain is protective against ischemic cardiac or neural injury, respectively (Kunze et al., 2012; Hölscher et al., 2011). Phd1 knockout mice have altered metabolism in skeletal muscles and overall enhanced hypoxia tolerance (Aragonés et al., 2008); and germline Phd3 knockout mice are hypotensive due to a hypofunctional sympathoadrenal system (Bishop et al., 2008).

There has been interest to identify non-HIF substrates for PHDs and other pathways that are regulated through PHD-catalyzed hydroxylation. In the last two decades, more than 20 non-HIF substrates for PHDs have been reported, and hypoxia-mediated hydroxylation of these proteins alters response of downstream pathways (Cockman et al., 2019; Strowitzki et al., 2019). Most of these substrates were identified through cellular studies, suggesting that PHDs may act upon non-HIF substrates under physiological conditions (Strowitzki et al., 2019; Zheng et al., 2014; Moser et al., 2013; Segura et al., 2016; Guo et al., 2016; Köditz et al., 2007; Xie et al., 2009; Ullah et al., 2017; Deschoemaeker et al., 2015; Rodriguez et al., 2018). One group found in in vitro enzymatic assays that PHDs lacked detectable activities on these non-HIF substrates. This difference in findings between cellular and in vitro enzymatic assays suggests that the action of PHDs on non-HIF substrates requires additional cellular machinery, such as adaptors or post-translational modifications (Cockman et al., 2019).

FIH1 is another 2-OG dioxygenase that is known to sense hypoxia and regulate the HIF pathway (Figure 2A; Lando et al., 2002a; Mahon et al., 2001). Under normoxia, FIH1 catalyzes the asparaginyl hydroxylation of the C-terminal transactivation domain (CTAD) of HIFα (Table 3; Koivunen et al., 2004; Lando et al., 2002b), which is responsible for its binding with the transcriptional coactivator p300/CBP (Lando et al., 2002b; Freedman et al., 2002). FIH1-catalyzed asparaginyl hydroxylation of the CTAD impairs the recruitment of p300/CBP and reduces transcriptional activity of HIF (Lando et al., 2002a; Lando et al., 2002b). In hypoxia, FIH1 activity is also reduced by hypoxia, enabling HIFα to recruit p300/CBP for transcriptional activation of its target genes (Lando et al., 2002a; Lando et al., 2002b). The reported O_2_ Km value for FIHs is 90 μM, using a HIF1α peptide containing site Asn803 (Table 4; Koivunen et al., 2004). Compared with the Km values of PHDs in similar assays, FIH1 appears to be less sensitive to hypoxia, that is, as O_2_ levels decrease, PHDs are inhibited before FIH1 (Tian et al., 2011). Thus, FIH1 is considered a fine modulator of the HIF pathway in sensing severe hypoxia. Consistent with the notion that its role is more limited, FIH1 knockout mice have abnormal metabolism but not other HIF-regulated processes (Zhang et al., 2010; Sim et al., 2018). There are also non-HIF substrates identified for FIH1 that may also be regulated in an O_2_-dependent manner (Table 3; Cockman et al., 2009b; Scholz et al., 2016; Cockman et al., 2009a).

#### Epigenetic modulators

These include the lysine demethylase (KDM) Jumonji C (JmjC) domain-containing proteins, and the DNA demethylases ten-eleven translocation enzymes (TETs).

JmjC domain-containing proteins contain domains for Fe(II) and 2-OG binding and catalytic activities (Shmakova et al., 2014; Kooistra and Helin, 2012). They also bind with O_2_ and utilize it as a substrate, therefore having the potential to function as hypoxia sensors if their O_2_-binding affinities allow (Shmakova et al., 2014). Of the 32 identified JmjC proteins in humans, at least 23 conduct lysine demethylation reactions (Shmakova et al., 2014). Their substrates include both histone lysines (K4, K9, K27, and K36 on histone 3) and some non-histone lysines. (Table 5). Histone methylations affect chromatin structure and compactness and consequently regulate gene expression in either activating or silencing mode (Table 5; Shmakova et al., 2014; Kooistra and Helin, 2012; Walport et al., 2016; Li et al., 2022). H3K4me2/3, H3K9me2, H3K27me3, and H3K36me3 levels increase after hypoxia, possibly due to KDMs acting as O_2_ sensors and effecting chromatin changes.

**Table 5.** JmjC domain-containing histone demethylases and their substrates*. (A = activating transcription, S = silencing transcription).

KDM class	Members (gene symbol)	Histone lysyl residue substrates	Other substrates
KDM2	KDM2A	H3K36me1/me2 (A)	p65, NF-κB
	KDM2B	H3K36me1/me2 (A), H3K4me3 (A)	
KDM3	KDM3A	H3K9me1/me2 (S)	PGC-1α K224me
	KDM3B	H3K9me1/me2 (S)	
	JJMJD1C	H3K9me1/me2 (S)	
KDM4	KDM4A	H3K9me2/me3 (S), H3K36me2 (A), H1.4K26me2/me3	WIZ, CDYL1, CSB, and G9a
	KDM4B	H3K9me2/me3 (S), H3K36me2 (A), H1.4K26me2/me3	WIZ, CDYL1, CSB, and G9a
	KDM4C	H3K9me2/me3 (S), H3K36me2 (A), H1.4K26me2/me3	WIZ, CDYL1, CSB, and G9a
	KDM4D	H3K9me2/me3 (S)	
	KDM4E	H3K9me3 (S)	H3R2me2/me1, H3R8me2/me1, H3R26me2/me1, H4R3me2
KDM5	KDM5A	H3K4me2/me3 (A)	
	KDM5B	H3K4me2/me3 (A)	
	KDM5C	H3K4me2/me3 (A)	H3R2me2/me1, H3R8me2, H4R3me2a, ULK1R170me2a
	KDM5D	H3K4me2/me3 (A)	
KDM6	KDM6A	H3K27me2/me3 (S)	
	KDM6B	H3K27me2/me3 (S)	
	KDM6C		
KDM7	KDM7A	H3K9me1/me2 (S), H3K27me1/me2 (S)	
	PHF8	H3K27me1/me2 (S), H4K20me1	
	PHF2	H3K9me2/me3 (S)	
Jmjc domain only	NO66	H3K4me2/me3 (A), H3K36me2/me3 (A)	Rpl8
	MINA53	H3K9me3 (S)	Rpl27a
	KDM8	H3K36me2 (A)	NFATc1
	JMJD6		H3R2me2,H4R3me2/me1, U2AF2/U2AF65, LUC7L2

* Known hydroxylation/demethylation sites are indicated.

KDM6A, also known as UTX, catalyzes demethylation at H3K27me2/me3 (Table 5; Hong et al., 2007). In 2019, Chakraborty et al. reported that increase in H3K27me3 levels during hypoxia is HIF-independent and is caused by the direct inhibition of KDM6A due to decreased pO_2_ under hypoxia (Figure 2A; Chakraborty et al., 2019). The O_2_ sensitivity of KDM6A was further confirmed by its O_2_ Km value of 180 μM (Table 4), in a similar range as the PHDs and FIH and the highest among KDM6 members (Chakraborty et al., 2019). Sensing of hypoxia by KDM6A can control cell fate by chromatin reprogramming (Chakraborty et al., 2019). For example, it is reported that in mouse myoblast C2C12 cells, increase in H3K27me3 levels due to inactivation of KDM6A represses the expression of myogenic genes and blocks myogenic differentiation (Chakraborty et al., 2019).

KDM5A catalyzes demethylation at H3K4me2/me3 (Table 5) and was recently reported by Batie et al. to be another hypoxia sensor that could directly regulate cell fate through chromatin reprogramming (Figure 2A; Christensen et al., 2007; Batie et al., 2019). KDM5A also has a relatively low O_2_ affinity, with a Km ~90 μM (Table 4; Batie et al., 2019). Inactivation of KDM5A by hypoxia results in rapidly increasing H3K4me3 levels, and downstream effects include active transcriptions of genes associated with antiproliferation, antiapoptosis, etc. (Batie et al., 2019). Excitingly, a new study found that KDM5A binding to H3K4me3 is enhanced by PHD1-mediated hydroxylation of H3 at proline residue 16 (H3P16OH) (Liu et al., 2022). This KDM5A-PHD1 axis raises the possibility of other cross-talk between O_2_ sensors.

KDM4 family enzymes mainly catalyze the demethylation at H3K9me2/me3 and H3K36me2 (Table 5; Hillringhaus et al., 2011). Their O_2_-binding affinities have also been investigated in vitro: the O_2_ Km values of KDM4A, KDM4B, KDM4C, and KDM4E are all within the range of 57–197 μM (Table 4; Cascella and Mirica, 2012; Hancock et al., 2017). These Km values suggest the potential of KDM4 members to be hypoxia sensors, but this depends on their cellular roles and the downstream responses of their speculated inhibition during hypoxia (Figure 2B). Of these KDM4 members, cellular activity of KDM4A is reported to show a graded response to O_2_ concentration in U2OS cells and so does the demethylation levels on H3K9me3 (Hancock et al., 2017). It is also reported that KDM4A regulates the transcription of HIF1α through the H3K9 methylation status at HIF1α locus during hypoxia in tumors (Dobrynin et al., 2017). More studies for the function of other KDM4 demethylases during hypoxia are still needed.

There are also cases where the JmjC-containing KDMs function as hypoxia sensors through non-histone substrates. One such example is KDM3A, a histone demethylase for H3K9me2/1 sites (Table 5), whose activity on H3K9me2 is maintained even under severe hypoxia (0.2% O_2_), suggesting a high binding affinity with O_2_ with this substrate (Yamane et al., 2006; Brauchle et al., 2013; Beyer et al., 2008). However, recently Qian et al. discovered that the demethylation activity of KDM3A on a non-histone substrate, peroxisome proliferator-activated receptor gamma coactivator (PGC-1α) K224me, is inhibited by hypoxia (Figure 2A), with Km ~7.6% O_2_ (~75 μM), high enough to function as a hypoxia sensor under physiological conditions (Table 4; Qian et al., 2019). PGC-1α is a transcriptional coactivator that binds with transcriptional factor nuclear respiratory factor (NRF1/2) for activating transcription of nucleus-encoded mitochondrial genes (Qian et al., 2019; Scarpulla et al., 2012). The inhibited activity of KDM3A causes the accumulation of K224 mono-methylation on PCG-1α, which reduces the interaction between PCG-1α and NRF1/2, decreasing mitochondrial biogenesis (Qian et al., 2019). Another example is KDM5C, a histone demethylase for H3K4me2/me3 sites, which can also function as an arginine demethylase (Figure 2A; Li et al., 2022; Iwase et al., 2007). Its demethylation of ULK1 R170me2s site is inhibited by 1% O_2_ level in LN229 and several other cell lines (Li et al., 2022). This inhibited demethylation stabilizes ULK1 R170me2s, which further activates ULK1 and induces autophagy as a downstream response (Li et al., 2022). The cases of KDM3A and KDM5C suggest the possibility of other JmjC-containing KDMs to sense and respond to hypoxia through undiscovered non-histone substrates.

Besides KDMs, another set of 2-OG-dependent dioxygenases that act as epigenetic regulators are the TET enzymes (TET1, TET2, and TET3 in humans). These enzymes catalyze the hydroxylation of DNA 5-methylcytosine (5mC) to 5-hydroxymethylcytosine (5hmC) (Ponnaluri et al., 2013; Wu and Zhang, 2017). This facilitates the subsequent demethylation of 5hmC into an unmodified cytosine (Ponnaluri et al., 2013; Wu and Zhang, 2017). Since CpG methylation is typically silencing, TETs tend to promote gene activation. A large variance exists in different reports about the O_2_ Km values of TET1 and TET2, ranging from 0.31% and 0.53% (3.0 μM and 5.2 μM) using genomic DNA as substrates to 30 μM using oligonucleotides as substrates (Table 4); however, these measurements show much tighter O_2_ binding of TET1 and TET2 compared with the aforementioned reported hypoxia sensors (KDM3A, KDM5A, and KDM6A) (Laukka et al., 2016; Thienpont et al., 2016). Severe hypoxia, such as 0.5% O_2_ treatment, is reported to directly impair the cellular activities of TETs, increase DNA hypermethylation, and decrease the expression levels of associated genes (Figure 2A; Thienpont et al., 2016). DNA hypermethylation caused by TETs inhibition also happens during pathophysiological hypoxia found in tumors (Thienpont et al., 2016). Considering their O_2_ sensitivity, TETs are more likely to function as hypoxia sensors under extreme hypoxic conditions.

#### Translational modulators

Translational modulators in the 2-OG-dependent dioxygenases include the mRNA hydroxylases and ribosome hydroxylases.

The most abundant RNA modification is N^6^-methylation of adenosine (m^6^A), which affects the processing, splicing, translation, and degradation of modified mRNAs (Zaccara et al., 2019; Chen et al., 2019). The dynamics of m^6^A modification is coordinated by methyltransferases (so-called ‘writers’), demethylases (‘erasers’), and identifiers (‘readers’) (Zaccara et al., 2019; Chen et al., 2019). Two m^6^A RNA demethylases have been identified thus far: FTO and ALKBH5, with demonstrated demethylase activity in vitro and in vivo, respectively (Zheng et al., 2013; Jia et al., 2011.Zaccara et al., 2019; Zheng et al., 2013; Mauer et al., 2017). Indirect evidence for a sensor role of these enzymes in hypoxia response is that despite their protein levels staying relatively constant, hypoxia leads to m^6^A accumulation in cancer cells and breast cancer. Thus, it is possible that hypoxia plays a role in direct inhibition of ALKBH5 and/or FTO (Figure 2B). More studies about other possible demethylases for m^6^A in mRNA and the O_2_-binding affinities of these enzymes are needed to determine which of them directly sense and respond to hypoxia.

The translational apparatus may itself be targeted by ribosome hydroxylases, which modify the histidyl or prolyl residues of ribosomal subunit proteins (Bundred et al., 2018; Zhuang et al., 2015). These ribosome hydroxylases regulate translation and participate in physiological or disease processes, including cellular growth, skeletal bone formation, tumorigenesis, and immune regulation (Bundred et al., 2018; Zhuang et al., 2015; Ge et al., 2012; Singleton et al., 2014). Currently, three ribosome hydroxylases have been identified: histidyl hydroxylases MINA53 (RIOX2) and NO66 (RIOX1) targeting the 60S large subunits Rpl27a and Rpl8, respectively; and prolyl hydroxylase OGFOD1 targeting the 40S small subunit Rpl23 (Ge et al., 2012; Singleton et al., 2014). Among these, NO66 has its activity inhibited by 0.1–1% O_2_ in cellular studies (Figure 2B). By contrast, OGFOD1 still retains 80% of its cellular activity even under severe hypoxia (0.2% O_2_) (Ge et al., 2012; Singleton et al., 2014). While this suggests the potential for NO66 to be a sensor in severe hypoxia, more studies of the O_2_ affinity of NO66 and the downstream response of its inhibition by hypoxia are needed.

#### Heme-dependent dioxygenases

Heme prosthetic groups are used by these enzymes for O_2_ binding and activation (Figure 3—figure supplement 2; Huang and Groves, 2018; Efimov et al., 2011; Raven, 2017). Five known heme-dependent dioxygenases in humans are indoleamine 2,3-dioxygenase (IDO) 1 and 2, tryptophan 2,3-dioxygenase (TDO), and prostaglandin G/H synthase (PGHS) 1 and 2 (Paton and Ntambi, 2009; Efimov et al., 2011; Raven, 2017). Except for IDO2 that has not been measured, these dioxygenases have reported Km values of 10–30 μM (Table 4; Juránek et al., 1999; Kolawole et al., 2015). As reflected by their lower O_2_ Km values, heme-dependent dioxygenases tend to have stronger O_2_ binding than 2-OG-dependent dioxygenases.

Both TDO and IDOs catalyze the conversion of L-tryptophan to N-formyl-L-kynurenine (Figure 3C; Thackray et al., 2011). They have similar heme- and substrate-binding pockets, although they share low sequence identity overall, and are believed to be an example of convergent evolution (Ball et al., 2014; Thackray et al., 2011). Both TDO and IDO regulate immune responses, possibly by modifying tryptophan homeostasis. TDO and IDO have distinct tissue expression patterns, with TDO mostly restricted to liver and epidermis, while IDO is found throughout the body and can be induced by certain immune or inflammation signals (Ball et al., 2014). Their expression patterns may further regulate their relative importance in different tissues or toward different stimuli (Ball et al., 2014). The cellular activity of TDO is reported to be inhibited by hypoxia (1–10% O_2_) in HeLa cells transfected with TDO, while TDO protein level remains unaltered (Elbers et al., 2016). Cellular activity of IDO1 is also decreased by hypoxia (1% O_2_) in 86HG39 and HeLa cells with unaltered IDO1 protein level (Schmidt et al., 2013). Impaired immune responses are observed in both cases as downstream effects (Figure 2B; Elbers et al., 2016; Schmidt et al., 2013). Further studies are needed to clarify whether hypoxia directly inhibits the enzymatic activities of TDO and IDO1 and how this might trigger downstream responses in a more physiological system.

#### Lipoxygenases

Lipoxygenases (LOXs) are iron-containing dioxygenases that catalyze the insertion of O_2_ into polyunsaturated fatty acids (PUFA) and their derivatives, forming hydroperoxyl eicosatetraenoic acid (HPETE) products (Figure 3D). HPETE products are chemically unstable and reduced by peroxidases to hydroxyl eicosatetraenoic acid (HETE) (Biringer, 2020; Ivanov et al., 2010; Kuhn et al., 2015). In humans, there are six known LOXs with arachidonic acid as the most common substrate (Biringer, 2020; Kuhn et al., 2015). These arachidonate lipoxygenases (ALOXs) are named according to the positional specificity in their catalyzed hydroperoxyl reactions as the ALOX5, ALOX12, ALOX12B, ALOX15, ALOX15B, and ALOXE3 (Biringer, 2020; Kuhn et al., 2015). Functions of ALOXs include biosynthesis of inflammatory mediators as well as regulation of cellular redox state (Kuhn et al., 2015).

ALOXs demonstrate that O_2_-binding affinities can be affected by the specific substrate, as illustrated by ALOX15. The reported O_2_ Km values of human ALOX12, rat ALOX5, and rabbit ALOX15 are all within the range of 8–26 μM, as measured by biochemical studies with arachidonic acid as the substrate (Table 4; Juránek et al., 1999; Wecksler et al., 2009). While the rabbit ALOX15 reaches its V_max_ under normoxia with arachidonic acid as the substrate, its reaction rate with hydroxyl arachidonic acids as substrates still increases with increasing O_2_ concentration under hyperoxic conditions (Ivanov et al., 2005). This suggests a higher O_2_ Km value, a lower O_2_-binding affinity, and the ability to sense the change of O_2_ concentration from normoxic to hypoxic conditions when hydroxyl arachidonic acids are the substrates for rabbit ALOX15. Similarly, human ALOX15 has O_2_ Km values for different substrates, namely 24 μM for arachidonic acid and 9.6 μM for linoleic acid. Furthermore, allosteric binding of 12-HEHE to ALOX15 affects its O_2_ affinity (Wecksler et al., 2009).

The fact that substrates affect O_2_ affinity is not a mere laboratory curiosity. Multiple ALOX substrates may be involved in hypoxia-related diseases, including pulmonary hypertension and cardiovascular diseases (Mashima and Okuyama, 2015; Zhu and Ran, 2012; Ivanov et al., 2015). Studying the substrate-dependent O_2_ sensitivity of human ALOXs in various biological contexts will be necessary to ascertain whether and how these enzymes sense and respond to hypoxia in vivo.

#### Other dioxygenases

Ten other human dioxygenases have been identified (Supplementary file 1). They have the shared property of using an octahedral Fe(II) as the catalytic center.

Among these 10 enzymes, cysteamine (2-aminoethanethiol) dioxygenase (ADO) has been identified as a hypoxia sensor (Masson et al., 2019). ADO catalyzes the oxidation of protein N-terminal cysteines to cysteine sulfinic acid (Figure 3E) and promotes the degradation of the oxidized substrate protein through the N-degron pathway (Masson et al., 2019). Human ADO has a relatively low O_2_-binding affinity (Km > 500 μM, Table 4, Masson et al., 2019). As a result, even mild hypoxia inhibits ADO activity, allowing stabilization of its substrates, including the regulator of G protein signaling (RGS4/5) and cytokine interleukin (IL)-32 (Masson et al., 2019). During hypoxia, inhibited ADO results in the stabilization of RGS4/5 and subsequently modulates G protein-coupled calcium ion signals and mitogen-activated protein kinase (MAPK) signaling (Figure 2A; Masson et al., 2019). Hypoxia sensing by ADO provides a faster response compared with HIF-mediated transcriptional regulation (Masson et al., 2019).

### Monooxygenases

The monooxygenase members can be further classified into iron-dependent, copper-dependent, and flavin-dependent monooxygenases based on their catalytic centers (Table 2).

#### Iron-dependent monooxygenases

Most monooxygenases utilize iron as the catalytic center for oxygen insertion and can be further divided into heme-dependent and non-heme-dependent ones.

#### Heme-dependent monooxygenases

In humans, these include cytochrome P450 enzymes, heme oxygenases, and nitric oxide synthases.

Cytochrome P450 enzymes (CYPs), which comprise ~60% of all human monooxygenases (Supplementary file 1), are responsible for the oxidative metabolism of both endogenous and exogenous chemicals (Guengerich, 2007; Danielson, 2002; Poulos and Johnson, 2005). They play an important role in the synthesis and metabolism of hormones, cholesterols, and vitamins, and the clearance and detoxification of xenobiotics (Danielson, 2002; Poulos and Johnson, 2005). Not only do they have diverse biological functions, but their biochemical properties vary widely as well. For instance, the substrate specificity of CYPs ranges from a single substrate (such as CYP19A1) to a diverse repertoire of substrates (Poulos and Johnson, 2005). The O_2_ sensitivities of CYPs have been assessed for drug clearance in cellular systems or subcellular systems such as liver microsomes (Fradette and Du Souich, 2004). With different drug substrates used in the assays, a wide range of O_2_ Km values of mixed CYPs have been reported, ranging from 0.5 to 200 μM in mammalian species (Jones, 1981). This is consistent with reports that hypoxia could increase the half-life and/or toxicity of certain drugs. However, it is currently unclear whether CYPs do, in fact, function as hypoxia sensors. Direct evidence is lacking that cellular CYP activity is affected by hypoxia, and their O_2_ Kms with endogenous substrates are largely unknown. It is known, however, that hypoxia affects the expression levels of some CYPs, suggesting that these CYPs may have a role in hypoxia response (Fradette and Du Souich, 2004). In principle, a given CYP could act as both a hypoxia sensor and downstream effector.

Heme oxygenases (HO) catalyze the degradation of cellular heme to biliverdin, also producing ferrous iron and carbon monoxide (Figure 3F; Yoshida and Migita, 2000). There are two catalytically active human heme oxygenases, HO-1 and HO-2. They do not contain prosthetic heme groups for their catalytic reactions; instead, they bind heme substrates that are used for O_2_ binding, activation, and reduction (Yoshida and Migita, 2000). Although their O_2_ Kms are unknown, their estimated dissociation constant (Kd) is 0.012–0.034 μM (Migita et al., 1998). These very high O_2_-binding affinities suggest that HOs are unlikely to be direct O_2_ sensors. Instead, the activity of HO-2 may indirectly be regulated by O_2_ through redox potential, which has implications for whole-body sensing in the carotid body (López-Barneo et al., 2008; Ragsdale and Yi, 2011), thereby affecting whole-body physiological response to hypoxia.

Nitric oxide synthases (NOSs) convert L-arginine into nitric oxide (NO) in two steps, each using one O_2_ molecule activated by the heme iron (Figure 3G; Daff, 2010). NO is a gas signaling molecule with an array of functions, including regulation of vascular tone, immune defense, neural development, and hypoxia signaling (Moncada and Higgs, 1991; Ho et al., 2012). There are three human NOS enzymes: neuronal NOS (NOS1 or nNOS) is constitutively expressed in nerve, skeletal muscle, and heart muscle cells; inducible NOS (NOS2 or iNOS) is induced in multiple immune cells after stimuli; and endothelial NOS (NOS3 or eNOS) is constitutively expressed in vascular endothelial cells (Daff, 2010).

The O_2_ Km values for all three NOSs have been reported and are quite different from each other: 350 μM for rat nNOS, 130 μM for mouse iNOS, and 4 μM for bovine eNOS when using L-Arg as substrate (Table 4; Stuehr et al., 2004; Santolini et al., 2001a; Abu-Soud et al., 2000; Abu-Soud et al., 2001; Santolini et al., 2001b; Abu-Soud et al., 1996). (Although measured from different species, these values have been compared with each other to illustrate the different O_2_ affinities of these three NOSs; Abu-Soud et al., 2001; Semenza, 2005.) These differences in O_2_ Km values, together with tissue-specific expression patterns of different NOS isoforms, account for their distinct roles in response to hypoxia. The low O_2_ Km value of eNOS may help eNOS enzymatic activity remain constant across O_2_ concentration ranges in vascular endothelial cells (Ho et al., 2012; Semenza, 2005). The high Km value of nNOS indicates its enzymatic activity is more dependent on O_2_ concentrations, and it is reported to have a linear relationship between O_2_ concentration and its NO-producing activity over the entire physiological O_2_ range (Santolini et al., 2001a; Semenza, 2005; Elayan et al., 2000). This suggests the activity of nNOS decreases during acute hypoxia, which should be neuroprotective as excessive NO is reported to increase neurotoxicity during acute ischemic stroke (Ho et al., 2012; Dawson and Dawson, 1996). In fact, hyperbaric O_2_ treatment can increase NO production in rat nNOS and lead to neurotoxicity, a hint that nNOS could also function to sense hyperoxia (Elayan et al., 2000). During chronic hypoxia, nNOS functions more as an effector: upregulation of nNOS expression level leads to the increase of NO production to increase blood flow by vasodilation (Ward et al., 2005). The Km value of iNOS is also high enough for a hypoxia sensor, but iNOS is not typically expressed and needs to be induced by different stimulus in most human tissues, making the condition for it to function as a sensor more complicated (Abu-Soud et al., 2001; Robinson et al., 2011).

In addition, the NOSs can also cross-talk with the PHD-HIF-pVHL pathway by NO-derived cysteine S-nitrosylation of HIF1α and pVHL protein, which can inhibit the binding between hydroxylated HIF1α and pVHL and stabilize HIF1α even when O_2_ is not limiting (Li et al., 2007; Palmer et al., 2007). This may play a role in immune cells where iNOS can be induced to activate HIF-mediated immune response (Li et al., 2007).

#### Non-heme Fe-dependent monooxygenases

There are eight identified non-heme Fe-dependent monooxygenases in humans (Supplementary file 1) that utilize several different cofactors for iron coordination. Five use (6R)-*L-*erythro-5,6,7,8-tetrahydrobiopterin (BH_4_) as an electron donor and co-substrate, namely tyrosine 3-hydroxylase (TH), tryptophan 5-hydroxylase 1 and 2 (TPH1 and TPH2), phenylalanine-4-hydroxylase (PAH), and alkylglycerol monooxygenase (Bassan et al., 2003; Watschinger et al., 2010). Similar to 2-OG-dependent dioxygenases, the catalytic iron is coordinated in an octahedral mode for binding and activation of O_2_ (Bassan et al., 2003).

TH catalyzes the hydroxylation of L-tyrosine into L-3,4-dihydroxyphenylalanine (L-DOPA) (Figure 3H), the rate-limiting step in biosynthesis of catecholamines (dopamine, noradrenaline, and adrenaline) (Rostrup et al., 2008). The O_2_ Km values of TH vary by splice isoform, ranging from 12 to 47 μM across the four splice isoforms as measured in in vitro enzymatic assays (Table 4; Rostrup et al., 2008; Katz, 1980). In cellular assays, the activity of TH in PC12 cells was inhibited when O_2_ concentration decreased from 139 to 33 μM (Rostrup et al., 2008). These measurements suggest that acute hypoxia inhibiting TH could lead to decreased synthesis of catecholamines (Figure 2B; Raghuraman et al., 2012; Souvannakitti et al., 2009). Since dopamine inhibits the chemotransduction of the carotid body in most mammals, suppression of TH activity during acute hypoxia may sensitize the carotid body to hypoxia (Iturriaga and Alcayaga, 2004; Iturriaga et al., 2009). In contrast to acute hypoxia, chronic or intermittent hypoxia leads to upregulation of TH activity via increased mRNA and/or phosphorylation, countering its decreased enzymatic activity (Kumar et al., 2003; Hui et al., 2003; Schnell et al., 2003).

PAH and TPH, like TH, are also aromatic amino acid hydroxylases, with similar structures and catalytic mechanisms (Bassan et al., 2003). The O_2_ Km values of human PAH and rat TPH are reported to be 17 uM and 3.9–12.9 uM in enzymatic assay, respectively (Table 4; Katz, 1980). Their potential for hypoxia sensing and responding needs further exploration.

#### Copper-dependent monooxygenases

Besides iron, copper is also frequently used for O_2_ binding and activation by oxidizing enzymes. In humans, there are five identified or speculated monooxygenases that use copper as the catalytic center (Supplementary file 1). These enzymes all have two copper ions at their active sites but employ different strategies for O_2_ binding and activation, depending on whether the two copper irons are in sufficient proximity to be magnetically coupled (Decker and Solomon, 2005; Lewis and Tolman, 2004). The coupled binuclear Cu enzymes such as tyrosinase (TYR) use both copper ions for O_2_ binding and activation, while the non-coupled binuclear Cu enzymes such as peptidylglycine α-amidating monooxygenase (PAM) and dopamine b-monooxygenase (DβM) use only one copper iron (Cu_B_) for this process (Decker and Solomon, 2005; Lewis and Tolman, 2004). The crystal structure of PAM shows that Cu_B_ has a tetrahedral structure, coordinated by two His residues and one Met residues, with the other position for O_2_ binding (Prigge et al., 2004).

PAM catalyzes the amidation of C-terminal glycines in peptides (Figure 3I), a post-translational modification that may affect substrate stability (Simpson et al., 2015). PAM activity is progressively inhibited from mild (7% O_2_) to severe (1% O_2_) hypoxia in mammalian cells (Figure 2B; Simpson et al., 2015). Rat PAM has been shown to have high O_2_ Km values (100–550 μM), with this wide range attributable to different degrees of substrate hydrophobicity (Table 4; McIntyre et al., 2010). The best-characterized substrates of PAM are endocrine peptides, for example, chromogranin A (CgA), whose amidation by PAM is profoundly suppressed by hypoxia (Simpson et al., 2015; Merkler, 1994). However, the functional consequence of this change in amidation remains unclear (Simpson et al., 2015).

#### Flavin-dependent monooxygenases

Flavin-dependent monooxygenases utilize a non-covalently bound FAD prosthetic group to activate O_2_ (Palfey and McDonald, 2010; Romero et al., 2018). Unlike above discussed O_2_-dependent enzymes whose reaction rates are saturated above an O_2_ threshold, reaction rates for these enzymes are thought to be directly proportional to O_2_ concentration. This suggests that decreased O_2_ concentration from normoxia to hypoxia could decrease reaction rates of these enzymes (Massey, 2002), although it is not clear how their cellular activities are affected by hypoxia.

### Oxidases

The oxidase members can be further classified into heme-copper, iron-dependent, copper-dependent, flavin-dependent, and other oxidases based on their catalytic centers (Table 2).

#### Heme-copper oxidases

Heme-copper oxidases (HCO) are the terminal oxidases in the aerobic respiratory chain that catalyze the 4-electron reduction of O_2_ to water (Ferguson-Miller and Babcock, 1996; Nolfi-Donegan et al., 2020). In mammals, this is the cytochrome c oxidase (CcO), also known as the Complex IV of the electron transport chain (ETC) in mitochondria (Nolfi-Donegan et al., 2020; Figure 3—figure supplement 3). Mammalian CcOs utilize a hetero-binuclear heme-copper center to activate O_2_ (Wikström et al., 2018; Namslauer and Brzezinski, 2004). O_2_ binds with both the heme iron and the copper as a ligand bridge and is then reduced with electrons passed from the reduced form of cytochrome c through other metal prosthetic sites (Wikström et al., 2018; Namslauer and Brzezinski, 2004; Aoyama et al., 2009). Compared with other O_2_-dependent enzymes, CcO has a high O_2_ affinity, with O_2_ Km values measured to be <1 μM in assays using intact cells or purified mitochondria with sufficient substrates (Table 4; Petersen et al., 1974; Bienfait et al., 1975; Gnaiger et al., 1995; Scandurra and Gnaiger, 2010). Based solely on its low Km values, CcO would not be expected to act as hypoxia sensors.

However, CcO appears to be inhibited during hypoxia (1–3% O_2_) (Chandel et al., 1997; Duranteau et al., 1998). Inhibition of CcO disrupts the ETC, which is related to electron leakage from Complex III and Complex I (Duranteau et al., 1998; Fuhrmann and Brüne, 2017; Hernansanz-Agustín et al., 2017; Guzy et al., 2007), increased mitochondrial produced ROS (Duranteau et al., 1998; Fuhrmann and Brüne, 2017; Hernansanz-Agustín et al., 2017; Guzy et al., 2007; Chandel et al., 2000), and altered downstream HIF, PI3K/Akt, AMPK, and MAPK signaling (Fuhrmann and Brüne, 2017; Guzy et al., 2007; Chandel et al., 2000; Brand, 2016; Kim et al., 2018; Emerling et al., 2005; Kulisz et al., 2002; Emerling et al., 2009). Thus, despite CcO’s low Km values it has hypoxia sensor-like properties. How CcO’s activities are regulated during hypoxia remains to be elucidated.

#### Iron-dependent oxidases

Iron-dependent oxidases include the desaturases and ferroxidases (Supplementary file 1; Jasniewski and Que, 2018). The structures and catalytic mechanisms are relatively poorly characterized, but the studied ones all have two Fe(II) ions, coordinated by five His/Glu residues from the enzymes, that bind and active O_2_ (Jasniewski and Que, 2018; Hess et al., 2010; Bertini et al., 2012).

Stearoyl-CoA desaturase 1 (SCD1) catalyzes the formation of the monounsaturated fatty acid oleic acid from the saturated fatty acid stearic acid (Figure 3J; Paton and Ntambi, 2009), thereby playing an important role in lipid metabolism, membrane fluidity, and cell integrity (Paton and Ntambi, 2009), as increased fatty acid saturation could result in lipotoxicity and cell death (Hess et al., 2010; Wang et al., 2006; Green and Olson, 2011). Inhibition of SCD1 activates the unfolded protein response (UPR) through ER stress (Green and Olson, 2011; Volmer et al., 2013). Although the O_2_ affinity of SCD1 is unknown, hypoxia (1% O_2_) impairs the cellular activity of SCD1 in A549 and HeLa cells, increasing the saturated fatty acid ratio and thereby altering the cellular lipid composition (Figure 2B; Kamphorst et al., 2013). SCD1 may well be a hypoxia sensor if the decrease in its activity is directly due to reduced O_2_ concentration.

#### Copper-dependent oxidases

Copper-dependent oxidases include the copper amine oxidases (CAOs) and lysyl oxidases (LOXs), both of which catalyze oxidative deamination of amines to the corresponding aldehydes, also producing hydrogen peroxide and ammonia (Figure 3K; Finney et al., 2014). Human CAO member AOC3 is reported to have an O_2_ Km ~38 μM in enzymatic assays (Table 4), and its cellular activity in adipocyte lysate is inhibited by hypoxia in a HIF-independent manner (Figure 2B; Shen et al., 2012; Repessé et al., 2015; Andrés et al., 2001; Morris et al., 1997). The cellular function of AOC3 is not clear since its endogenous substrates are unknown, although in vitro kinetics studies suggest dopamine and cysteamine as potential substrates (Shen et al., 2012).

#### Flavin-dependent oxidases

Similar to flavin-dependent monooxygenases, this group of oxidases utilize either FAD or FMN for the activation of O_2_ and have reaction rates proportional to the O_2_ concentration (Massey, 2002). The >20 members of this group (Table 2, Supplementary file 1) in humans have substrates ranging from small molecules (e.g., fatty acids and amino acids) to protein residues (Romero et al., 2018). Their role in hypoxia sensing and responding is unknown.

#### Other oxidases

Several other oxidases remain poorly characterized (Supplementary file 1). The dual oxidases DUOX1 and DUOX2 catalyze the formation of H_2_O_2_ from O_2_ molecules with electrons provided by NADPH (Donkó et al., 2005). In HIF1-deficient *Caenorhabditis elegans*, hypoxia-induced extracellular matrix (ECM) remodeling could be phenocopied by inactivation of BLI-3, the ortholog of human DUOXs. This suggests a potential role of BLI-3 as a hypoxia sensor independent from the HIF pathway (Figure 2B; Vozdek et al., 2018) and raises the possibility that human DUOXs also sense and respond to hypoxia in an HIF-independent manner.

#### ODE as hypoxia sensors in other organisms

ODEs are evolutionary ancient. The major emergence of ODEs occurred at the separation of terrestrial and marine bacteria, coinciding with the emergence of oxygenic photosynthesis ~3.1 billion years ago (Jabłońska and Tawfik, 2021). Given the importance of hypoxia sensing, the evolutionary conservation of the HIF pathway across metazoans comes as no surprise. HIF1α and PHD2 (EGLN1 in *C. elegans*) emerged early in evolution, whereas additional HIFα and PHD isoforms emerged later in more complex organisms as context-dependent and fine-tuned hypoxia sensing became necessary (Taylor and McElwain, 2010).

What about plants? Plants have a hypoxia sensor, ADO, shared with metazoans. ADO is a thiol dioxygenase that modulates Arg/N-degron pathways and was found to be a sensor in both humans and *Arabidopsis thaliana* (Masson et al., 2019). Besides ADO, plant cysteine oxidases (PCOs), homologs of ADO, also function as hypoxia sensors, regulating Arg/N-degron pathways in plants (White et al., 2017; Weits et al., 2014; White et al., 2018).

What about single-celled organisms? In fission yeast, two hypoxia-sensing mechanisms exist that converge on activation of Sre1 (the yeast SREBP homolog), a transcription factor that triggers a downstream hypoxia response. One mechanism is that hypoxia inhibits multiple ODEs that are required for sterol synthesis, and this suppression of sterol synthesis stimulates the cleavage (and hence activation) of Sre1 (Hughes et al., 2005). The other mechanism is that hypoxia inhibits Ofd1, a yeast prolyl 4-hydroxylase-like 2-OG-dependent dioxygenase. Similar to how PHD inhibition allows HIF1α stabilization, Ofd1 inhibition allows Sre1 stabilization (Hughes and Espenshade, 2008). Protozoa also have prolyl hydroxylases that regulate the stability of S-phase kinase-associated protein 1 (Skp1) and alter the cell cycle (Xu et al., 2012).

What about prokaryotes? Prokaryotes also sense O_2_, although they do not appear to use ODEs as sensors. In nitrogen-fixing bacteria (*Rhizobium meliloti*), changes in O_2_ levels impact the kinase activity of FixL, which phosphorylates the transcription factor FixJ to regulate the expression of nitrogen-fixing genes (Monson et al., 1995; Agron et al., 1994). In this case, O_2_ actually acts as an allosteric binding cofactor that leads to a conformation change of FixL (Monson et al., 1995). Hence, FixL directly interacts with O_2_ molecules, but O_2_ is not used as a substrate. In most of all of the above, although the details may differ, the key criteria for a hypoxia sensor are met: (1) direct interaction with O_2_, (2) utilization of O_2_ as a substrate except in the case of FixL, and (3) causing a downstream response.

### Connection of O_2_-dependent enzymes to hypoxia adaptations and diseases

Hypoxia is related to many diseases. Decreased O_2_ at high altitudes can lead to systemic, organismal-level hypoxia and induce acute and chronic mountain sickness (AMC, CMC) (Roach and Hackett, 2001; Villafuerte and Corante, 2016). Systemic hypoxia is also seen in some respiratory diseases and anemic conditions that have disruption in O_2_ uptake or transport (Lee et al., 2019). Ischemia resulting from the blockage of blood flow leads to cell death and failure of affected tissues, most notably heart (in myocardial infarction) and brain (in ischemic stroke). (Lee et al., 2019) However, other tissues can also be affected, including the intestine, kidney, and skeletal muscle.

As previously mentioned, many O_2_-dependent enzymes are regulated at a transcriptional, translational, and/or post-translational level in response to hypoxia. Depending on the specific downstream responses and cellular context, a change in enzymatic activity may confer protection or further injury in hypoxia. One of the greatest challenges in understanding the effects of hypoxia is discerning the effects of adaptation and hypoxia tolerance versus maladaptation and hypoxia-mediated tissue injury. Injury and adaptation almost always overlap, either in time, development, or tissue domains.

We conclude below with two scenarios illustrating the role of O_2_-dependent enzymes in hypoxia adaptation or diseases: (1) positively selected genetic adaptations associated with O_2_-dependent enzymes in high-altitude populations; and (2) mutations in genes targeted by drugs associated with O_2_-dependent enzymes for hypoxia-related diseases.

#### O_2_-dependent enzymes in hypoxia adaptations of high-altitude populations

Tibetan, Andean, and Ethiopian populations reside at altitudes above 3500 m with a decreased O_2_ pressure (<60% of sea level) due to hypobaric hypoxia. Distinct genetic adaptations and physiological characteristics have developed within each population to promote survival at altitude (Beall, 2006). These three groups of humans have resided at high altitude for different lengths of time: Andeans for 10,000–15,000 years (Aldenderfer, 2003), Tibetans for over 30,000 years (Qi et al., 2013), and Ethiopians for even longer (Alkorta-Aranburu et al., 2012).

In lowlanders, one of the major adaptations upon exposure to hypoxia is increased red blood cell production (erythropoiesis) (Windsor and Rodway, 2007). Acutely, the increased hemoglobin helps compensate for decreased blood pO_2_, but in the long run, this increases blood viscosity and thereby increases risk of blood clots and ischemia (Braekkan et al., 2010; Parati et al., 2018). Erythropoiesis during exposure to hypoxia is mainly regulated by EPO, a downstream target of HIF that regulates erythropoiesis by activating EPO receptors on erythroid progenitors in the bone marrow (Haase, 2013).

Do Tibetan highlanders maintain higher levels of hemoglobin compared to populations living at sea level? Somewhat surprisingly, their hemoglobin levels are actually similar to those of lowlanders (Beall et al., 1998; Beall et al., 1997), but they have increased vasodilation and blood flow to compensate for O_2_ delivery to tissues. Genetic studies have identified variants under positive selection at the *EGLN1 (PHD2*) and *EPAS1 (HIF2A*) loci (Haase, 2013; Lorenzo et al., 2014; Bigham et al., 2010; Yi et al., 2010; Simonson et al., 2010; Xu et al., 2011; Yang et al., 2017; Peng et al., 2011; Wuren et al., 2014). One variant of EGLN1 exhibits a lower O_2_ Km value, which enhances HIF degradation under hypoxia and contributes to blunting of EPO-mediated erythropoiesis (Lorenzo et al., 2014).

Interestingly, positive selection is also observed at the *EGLN1* and *EGLN2* loci in Andean populations, but unlike Tibetans, Andeans have higher hemoglobin levels than lowlanders (Beall et al., 1998; Bigham et al., 2010; Bigham et al., 2009). Genetic studies suggest that Andeans cope with the risks of augmented erythropoiesis by enhancing cardiovascular function associated with positive selection in *BRINP3*, *NOS2*, and *TBX5* (Crawford et al., 2017). Among these, NOS2, also known as iNOS (discussed above in the sensor section), synthesizes NO as a gas signaling molecule to modulate vascular tone upon induction of this gene (Moncada and Higgs, 1991; Robinson et al., 2011). How these gene variants mechanistically lead to adaptation awaits further study.

Ethiopians have a distinct adaptation pattern compared with Tibetans and Andeans. Some studies suggest that they maintain both normal blood saturation and hemoglobin concentration (Beall et al., 2002). A genome-wide scan identified HIF pathway-related genes, *ARNT2* and *THRB*, as candidate genes for positive selection with potential roles in the physiological response to hypoxia (Scheinfeldt et al., 2012).

Apart from these relatively well-studied HIF pathway-related genes, there are multiple other genes harboring variants associated with positive selection in these three highlander populations (Table 6). Notably, these include other known hypoxia sensors, such as *HIF1AN* and *KDM5A*, as well as potential hypoxia sensors, including *KDM4A*, *HMOX4*, *SCD,* and *DUOX2*. It will be interesting to further explore how these positively selected variants enhance hypoxia adaptation of highlanders.

**Table 6.** O_2_-dependent enzymes encoded by genes associated with positive selection in different high-altitude populations.

Population	Genes*
Tibetan	EGLN1 (Lorenzo et al., 2014; Bigham et al., 2010; Yi et al., 2010; Simonson et al., 2010; Xu et al., 2011; Yang et al., 2017; Peng et al., 2011; Wuren et al., 2014), CYP2E1 (Simonson et al., 2010), HMOX2 (Simonson et al., 2010; Peng et al., 2011; Wuren et al., 2014), CYP17A1 (Simonson et al., 2010; Wuren et al., 2014), SCD (Simonson et al., 2010), HIF1AN (Simonson et al., 2010), SC5D (Simonson et al., 2010), KDM5A (Simonson et al., 2010), HPD (Simonson et al., 2010), DOHH (Simonson et al., 2010), XDH (Simonson et al., 2010), CYP20A1 (Simonson et al., 2010), TMEM189 (Simonson et al., 2010), KDM4A (Simonson et al., 2010), PAOX (Simonson et al., 2010)
Andean	EGLN1 (Bigham et al., 2010; Bigham et al., 2009), EGLN2 (Bigham et al., 2009), NOS1 (Bigham et al., 2009), NOS2 (Bigham et al., 2010; Bigham et al., 2009; Crawford et al., 2017), DUOX2 (Jacovas et al., 2018), CYP39A1 (Eichstaedt et al., 2014), KDM2A (Eichstaedt et al., 2014), KMO (Eichstaedt et al., 2014), PLOD3 (Eichstaedt et al., 2014), P3H3 (Eichstaedt et al., 2014), CPOX (Eichstaedt et al., 2014), CYP24A1 (Eichstaedt et al., 2014)
Ethiopian	PCYOX1 (Scheinfeldt et al., 2012)

* Known hypoxia sensors are highlighted in red.

#### Pathogenic mutations and drug targets within O_2_-dependent enzymes for hypoxia-related diseases

Erythrocytosis commonly results from exposure to hypoxia. Genetic mutations in the pathway regulating erythropoiesis can also cause pathogenic erythrocytosis with excessive blood viscosity. Such pathogenic mutations have been found in genes, including (1) *VHL*, *EGLN1* (*PHD2*), and *EPAS1* (*HIF2A*) that affect EPO production, (2) *EPOR* and its regulator *JAK2* that affect erythroid progenitor maturation, and (3) hemoglobin subunits *HBA* and *HBB* that affect O_2_ delivery and tissue pO_2_ (Bento, 2018). Specifically, for *EGLN1*, more than 10 variants have been associated with erythrocytosis onset (Gardie et al., 2014). For example, one such mutation (P317R) has significantly decreased enzymatic activity (Percy et al., 2006). No mutations associated with pathogenic erythrocytosis have been identified in *EGLN2* and *EGLN3*, consistent with the notion that *EGLN1/PHD2* is the major isoform involved in HIF-mediated EPO upregulation.

Conversely, chronic kidney diseases (CKDs) lead to diminished EPO production and anemia. In adults, EPO is mainly produced by erythropoietin-producing cells (EPCs) in the kidney (Haase, 2013). The dysfunction of EPCs during CKDs results in EPO deficiency and is a key factor leading to associated anemia (Koury and Haase, 2015). Injectable erythropoiesis-stimulating agents (ESAs), such as recombinant human erythropoietin (rhEPO), are a cornerstone of CKD treatment (Singh et al., 2006; Pfeffer et al., 2009; Portolés et al., 2021). In recent years, PHD inhibitors have been developed as an alternative route to HIF stabilization and subsequent EPO production (Portolés et al., 2021; Gupta and Wish, 2017). Unlike rhEPO, PHD inhibitors ameliorate not only EPO deficiency but also inflammation and altered iron metabolism in CKD, both of which are regulated by HIF (Koury and Haase, 2015; Portolés et al., 2021). Currently, four PHD inhibitors (Roxadustat, Vadadustat, Daprodustat, and Molidustat) have entered or completed phase III clinical trials for treatment of the anemia of CKD (Portolés et al., 2021). Of them, Roxadustat and Daprodustat have been approved for use in Japan and/or China (Dhillon, 2019; Dhillon, 2020).

#### Open questions for discovering hypoxia sensors within the ODE members

Although we have an in-depth understanding of a small handful of O_2_ sensors, the potential landscape of hypoxia sensing in humans remains largely uncharted. Even once an ODE is confirmed to be a hypoxia sensor, much remains to be investigated.

#### At the enzymatic level

Most reported O_2_ Km values for ODEs are based on in vitro testing of the enzyme. However, in vivo, O_2_ Km depends on (1) the substrate (e.g., as discussed for ALOX12), and (2) the regulation of the ODE by other proteins and cofactors (e.g., as discussed for HO-2). Regarding (1), for a given ODE, what are its O_2_ affinities when catalyzing reactions using its various endogenous substrates in vivo? Answering this question requires identifying the in vivo substrates and then measuring O_2_ Km for each substrate. Regarding (2), how is the O_2_ Km of the ODE affected by modifying factors (e.g., PTMs and cofactor binding)? Answering this question requires identifying the modifying factors and then measuring O_2_ Km in the appropriate cellular contexts.

#### At the cellular level

Most studies of ODEs have focused on individual pathways directly responsible for downstream effects. However, these pathways do not act in isolation but rather as part of a network. Each ODE could have roles in multiple downstream response pathways, feedback loops, and cross-talk with other pathways. Ultimately, a systems-level understanding is needed to capture the complexity of O_2_ sensing within the cell.

#### At the tissue level

Currently, most studies of ODEs use cell models in which the O_2_ level is set to a single level controlled experimentally. However, this ignores the fact that from tissue-to-tissue, O_2_ levels vary substantially even at baseline. Furthermore, when an organism is exposed to hypoxic stress, the O_2_ levels from tissue-to-tissue and within a tissue can vary even further due to tissue-level changes such as vasodilation. This raises the question: what is the tissue specificity (and/or cell type specificity) of hypoxia sensors under basal and stressed conditions? An intriguing possibility is that each tissue might have a unique set of ODEs in order to sense and respond to the ongoing fluctuations in O_2_ concentration during maintenance of homeostasis, in accordance with tissue-specific O_2_ levels.

#### At the organismal level

Although an impressive diversity of molecular O_2_ sensors has been identified, their role in organismal-level adaptations to hypoxia remains unexplored territory. By far the best studied system for hypoxia sensing and response at the organismal level is PHD-HIF-pVHL pathway. Its role in improving O_2_ transport by increasing EPO synthesis by the kidney is well understood and has been the subject of many reviews (Haase, 2013; Nangaku and Eckardt, 2007).

There are numerous other adaptations to hypoxia that are much less well understood than HIF adaptations. An important one is the regulation of breathing. The mystery in this fundamental adaptation is the basis for gradually increasing breathing volume with time at altitude, such that blood O_2_ level is restored toward normal. This respiratory adaptation has several different time domains, and each likely has a unique set of sensors and effectors. O_2_ sensing at the carotid body is the first part of this response, and carotid body chemoreceptors have been the target of numerous attempts to identify molecular O_2_ sensors and the transduction pathways involved (López-Barneo et al., 2008). O_2_-sensitive potassium channels, redox sensors, and others have been proposed. Final agreement on the nature of the O_2_ sensor remains surprisingly elusive, perhaps reflecting that a diversity of O_2_ sensors take part in shaping the breathing response, not just one.

One of the challenges in linking molecular O_2_ sensors to responses at the organismal level is that organismal-level responses and adaptations are diverse. Vertebrate animals vary enormously on their tolerance of O_2_ deprivation. Some vertebrates, such as the crucian carp and the Western painted turtle, can survive for months without O_2_ (Bickler and Buck, 2007). These animals exceed the hypoxia tolerance of humans by a factor of at least 10,000 (Bickler and Buck, 2007). The molecular switches that orchestrate this impressive capability remain poorly defined. Certainly, if one is searching for molecular O_2_sensors, animals such as the carp and turtle would be fertile ground.

#### At the developmental level

Development as a model for changing O_2_ sensing and response has been little explored. Changes in O_2_ during development can be dramatic: the intrauterine environment of placental gas exchange has been likened to that of ascent of Mt. Everest, with a rapid increase in O_2_ upon aerial respiration at birth (Barcroft, 1946; Martin et al., 2010). The changes in O_2_ availability may signal crucial changes in synaptic physiology in the brain. How O_2_ sensing is regulated throughout development in accordance with changes in O_2_ levels is an important question to be answered.

### Summary

Aerobic organisms have evolved mechanisms to sense and respond to changes in O_2_ levels. O_2_ participates in hundreds of biochemical reactions regulating diverse, essential cellular processes. The enzymes responsible for these reactions directly interact with O_2_ and may function as hypoxia sensors by transducing the signal of low O_2_ via a decrease in enzymatic activity (rate or product yield). Here, we summarized and discussed the known and potential hypoxia sensors within each subcategory of O_2_-dependent enzymes in human, expanding from the well-known PHD enzymes, to the more recently identified sensors within the KDM family, to other enzymes with emerging roles in hypoxia sensing. We also discussed O_2_-dependent enzymes involved in hypoxia-related evolutionary adaptations and diseases, highlighting their relevance beyond chemical reactions. Much remains to be explored for most O_2_-dependent enzymes and roles in hypoxia. Are there new hypoxia sensors still to be discovered within O_2_-dependent enzymes? How do various hypoxia sensors coordinate with each other to regulate downstream cellular responses? What is the mechanism for each tissue to set its own hypoxia sensing threshold based on the specific physiological pO_2_? Furthermore, how can these discoveries help with hypoxia adaptation and disease treatment? All these questions await future research.

## Additional files

Supplementary file 1. Detailed information of 221 oxygen-dependent enzymes in human.

## References

[bib1] Abu-Omar MM, Loaiza A, Hontzeas N (2005). Reaction mechanisms of mononuclear non-heme iron oxygenases. Chemical Reviews.

[bib2] Abu-Soud HM, Rousseau DL, Stuehr DJ (1996). Nitric oxide binding to the heme of neuronal nitric-oxide synthase links its activity to changes in oxygen tension. Journal of Biological Chemistry.

[bib3] Abu-Soud HM, Ichimori K, Presta A, Stuehr DJ (2000). Electron transfer, oxygen binding, and nitric oxide feedback inhibition in endothelial nitric-oxide synthase. The Journal of Biological Chemistry.

[bib4] Abu-Soud HM, Ichimori K, Nakazawa H, Stuehr DJ (2001). Regulation of inducible nitric oxide synthase by self-generated NO. Biochemistry.

[bib5] Agron PG, Monson EK, Ditta GS, Helinski DR (1994). Oxygen regulation of expression of nitrogen fixation genes in Rhizobium meliloti. Research in Microbiology.

[bib6] Aldenderfer MS (2003). Moving up in the world: archaeologists seek to understand how and when people came to occupy the Andean and Tibetan plateaus. American Scientist.

[bib7] Alkorta-Aranburu G, Beall CM, Witonsky DB, Gebremedhin A, Pritchard JK, Di Rienzo A (2012). The genetic architecture of adaptations to high altitude in Ethiopia. PLOS Genetics.

[bib8] Andrés N, Lizcano JM, Rodríguez MJ, Romera M, Unzeta M, Mahy N (2001). Tissue activity and cellular localization of human semicarbazide-sensitive amine oxidase. The Journal of Histochemistry and Cytochemistry.

[bib9] Aoyama H, Muramoto K, Shinzawa-Itoh K, Hirata K, Yamashita E, Tsukihara T, Ogura T, Yoshikawa S (2009). A peroxide bridge between Fe and Cu ions in the O2 reduction site of fully oxidized cytochrome c oxidase could suppress the proton pump. PNAS.

[bib10] Appelhoff RJ, Tian Y-M, Raval RR, Turley H, Harris AL, Pugh CW, Ratcliffe PJ, Gleadle JM (2004). Differential function of the prolyl hydroxylases PHD1, PHD2, and PHD3 in the regulation of hypoxia-inducible factor. The Journal of Biological Chemistry.

[bib11] Aragonés J, Schneider M, Van Geyte K, Fraisl P, Dresselaers T, Mazzone M, Dirkx R, Zacchigna S, Lemieux H, Jeoung NH, Lambrechts D, Bishop T, Lafuste P, Diez-Juan A, Harten SK, Van Noten P, De Bock K, Willam C, Tjwa M, Grosfeld A, Navet R, Moons L, Vandendriessche T, Deroose C, Wijeyekoon B, Nuyts J, Jordan B, Silasi-Mansat R, Lupu F, Dewerchin M, Pugh C, Salmon P, Mortelmans L, Gallez B, Gorus F, Buyse J, Sluse F, Harris RA, Gnaiger E, Hespel P, Van Hecke P, Schuit F, Van Veldhoven P, Ratcliffe P, Baes M, Maxwell P, Carmeliet P (2008). Deficiency or inhibition of oxygen sensor Phd1 induces hypoxia tolerance by reprogramming basal metabolism. Nature Genetics.

[bib12] Baik AH, Jain IH (2020). Turning the oxygen dial: Balancing the highs and lows. Trends in Cell Biology.

[bib13] Ball HJ, Jusof FF, Bakmiwewa SM, Hunt NH, Yuasa HJ (2014). Tryptophan-catabolizing enzymes - party of three. Frontiers in Immunology.

[bib14] Barcroft J (1946). Researches on Pre-Natal Life.

[bib15] Bassan A, Blomberg MRA, Siegbahn PEM (2003). Mechanism of dioxygen cleavage in tetrahydrobiopterin-dependent amino acid hydroxylases. Chemistry.

[bib16] Batie M, Frost J, Frost M, Wilson JW, Schofield P, Rocha S (2019). Hypoxia induces rapid changes to histone methylation and reprograms chromatin. Science.

[bib17] Beall CM, Strohl KP, Blangero J, Williams-Blangero S, Almasy LA, Decker MJ, Worthman CM, Goldstein MC, Vargas E, Villena M, Soria R, Alarcon AM, Gonzales C (1997). Ventilation and hypoxic ventilatory response of Tibetan and Aymara high altitude natives. American Journal of Physical Anthropology.

[bib18] Beall CM, Brittenham GM, Strohl KP, Blangero J, Williams-Blangero S, Goldstein MC, Decker MJ, Vargas E, Villena M, Soria R, Alarcon AM, Gonzales C (1998). Hemoglobin concentration of high-altitude Tibetans and Bolivian Aymara. American Journal of Physical Anthropology.

[bib19] Beall CM, Decker MJ, Brittenham GM, Kushner I, Gebremedhin A, Strohl KP (2002). An Ethiopian pattern of human adaptation to high-altitude hypoxia. PNAS.

[bib20] Beall CM (2006). Andean, Tibetan, and Ethiopian patterns of adaptation to high-altitude hypoxia. Integrative and Comparative Biology.

[bib21] Bento C (2018). Genetic basis of congenital erythrocytosis. International Journal of Laboratory Hematology.

[bib22] Berra E, Benizri E, Ginouvès A, Volmat V, Roux D, Pouysségur J (2003). HIF prolyl-hydroxylase 2 is the key oxygen sensor setting low steady-state levels of HIF-1alpha in normoxia. The EMBO Journal.

[bib23] Bertini I, Lalli D, Mangani S, Pozzi C, Rosa C, Theil EC, Turano P (2012). Structural insights into the ferroxidase site of ferritins from higher eukaryotes. Journal of the American Chemical Society.

[bib24] Beyer S, Kristensen MM, Jensen KS, Johansen JV, Staller P (2008). The histone demethylases JMJD1A and JMJD2B are transcriptional targets of hypoxia-inducible factor HIF. The Journal of Biological Chemistry.

[bib25] Bickler PE, Donohoe PH (2002). Adaptive responses of vertebrate neurons to hypoxia. The Journal of Experimental Biology.

[bib26] Bickler PE, Buck LT (2007). Hypoxia tolerance in reptiles, amphibians, and fishes: life with variable oxygen availability. Annual Review of Physiology.

[bib27] Bienfait HF, Jacobs JM, Slater EC (1975). Mitochondrial oxygen affinity as a function of redox and phosphate potentials. Biochimica et Biophysica Acta.

[bib28] Bigham AW, Mao X, Mei R, Brutsaert T, Wilson MJ, Julian CG, Parra EJ, Akey JM, Moore LG, Shriver MD (2009). Identifying positive selection candidate loci for high-altitude adaptation in Andean populations. Human Genomics.

[bib29] Bigham A, Bauchet M, Pinto D, Mao X, Akey JM, Mei R, Scherer SW, Julian CG, Wilson MJ, López Herráez D, Brutsaert T, Parra EJ, Moore LG, Shriver MD, Begun DJ (2010). Identifying signatures of natural selection in Tibetan and Andean populations using dense genome scan data. PLOS Genetics.

[bib30] Biringer RG (2020). The enzymology of human eicosanoid pathways: the lipoxygenase branches. Molecular Biology Reports.

[bib31] Bishop T, Gallagher D, Pascual A, Lygate CA, de Bono JP, Nicholls LG, Ortega-Saenz P, Oster H, Wijeyekoon B, Sutherland AI, Grosfeld A, Aragones J, Schneider M, van Geyte K, Teixeira D, Diez-Juan A, Lopez-Barneo J, Channon KM, Maxwell PH, Pugh CW, Davies AM, Carmeliet P, Ratcliffe PJ (2008). Abnormal sympathoadrenal development and systemic hypotension in PHD3-/- mice. Molecular and Cellular Biology.

[bib32] Braekkan SK, Mathiesen EB, Njølstad I, Wilsgaard T, Hansen J-B (2010). Hematocrit and risk of venous thromboembolism in a general population: the Tromso study. Haematologica.

[bib33] Brand MD (2016). Mitochondrial generation of superoxide and hydrogen peroxide as the source of mitochondrial redox signaling. Free Radical Biology & Medicine.

[bib34] Brauchle M, Yao Z, Arora R, Thigale S, Clay I, Inverardi B, Fletcher J, Taslimi P, Acker MG, Gerrits B, Voshol J, Bauer A, Schübeler D, Bouwmeester T, Ruffner H (2013). Protein complex interactor analysis and differential activity of KDM3 subfamily members towards H3K9 methylation. PLOS ONE.

[bib35] Bugg TD (2001). Oxygenases: mechanisms and structural motifs for O(2) activation. Current Opinion in Chemical Biology.

[bib36] Bundred JR, Hendrix E, Coleman ML (2018). The emerging roles of ribosomal histidyl hydroxylases in cell biology, physiology and disease. Cellular and Molecular Life Sciences.

[bib37] Burmester T, Hankeln T (2014). Function and evolution of vertebrate globins. Acta Physiologica.

[bib38] Carreau A, El Hafny-Rahbi B, Matejuk A, Grillon C, Kieda C (2011). Why is the partial oxygen pressure of human tissues a crucial parameter? Small molecules and hypoxia. Journal of Cellular and Molecular Medicine.

[bib39] Cascella B, Mirica LM (2012). Kinetic analysis of iron-dependent histone demethylases: α-ketoglutarate substrate inhibition and potential relevance to the regulation of histone demethylation in cancer cells. Biochemistry.

[bib40] Chakraborty AA, Laukka T, Myllykoski M, Ringel AE, Booker MA, Tolstorukov MY, Meng YJ, Meier SR, Jennings RB, Creech AL, Herbert ZT, McBrayer SK, Olenchock BA, Jaffe JD, Haigis MC, Beroukhim R, Signoretti S, Koivunen P, Kaelin WG (2019). Histone demethylase KDM6A directly senses oxygen to control chromatin and cell fate. Science.

[bib41] Chandel NS, Budinger GR, Choe SH, Schumacker PT (1997). Cellular respiration during hypoxia: role of cytochrome oxidase as the oxygen sensor in hepatocytes. The Journal of Biological Chemistry.

[bib42] Chandel NS, McClintock DS, Feliciano CE, Wood TM, Melendez JA, Rodriguez AM, Schumacker PT (2000). Reactive oxygen species generated at mitochondrial complex III stabilize hypoxia-inducible factor-1alpha during hypoxia: a mechanism of O2 sensing. The Journal of Biological Chemistry.

[bib43] Chandrasekharan NV, Simmons DL (2004). The cyclooxygenases. Genome Biology.

[bib44] Chen XY, Zhang J, Zhu JS (2019). The role of m6A RNA methylation in human cancer. Molecular Cancer.

[bib45] Christensen J, Agger K, Cloos PAC, Pasini D, Rose S, Sennels L, Rappsilber J, Hansen KH, Salcini AE, Helin K (2007). RBP2 belongs to a family of demethylases, specific for tri-and dimethylated lysine 4 on histone 3. Cell.

[bib46] Cigognini D, Gaspar D, Kumar P, Satyam A, Alagesan S, Sanz-Nogués C, Griffin M, O’Brien T, Pandit A, Zeugolis DI (2016). Macromolecular crowding meets oxygen tension in human mesenchymal stem cell culture - A step closer to physiologically relevant in vitro organogenesis. Scientific Reports.

[bib47] Cockman ME, Webb JD, Kramer HB, Kessler BM, Ratcliffe PJ (2009a). Proteomics-based identification of novel factor inhibiting hypoxia-inducible factor (FIH) substrates indicates widespread asparaginyl hydroxylation of ankyrin repeat domain-containing proteins. Molecular & Cellular Proteomics.

[bib48] Cockman ME, Webb JD, Ratcliffe PJ (2009b). FIH-dependent asparaginyl hydroxylation of ankyrin repeat domain-containing proteins. Annals of the New York Academy of Sciences.

[bib49] Cockman ME, Lippl K, Tian YM, Pegg HB, Figg WD, Abboud MI, Heilig R, Fischer R, Myllyharju J, Schofield CJ, Ratcliffe PJ (2019). Lack of activity of recombinant HIF prolyl hydroxylases (PHDs) on reported non-HIF substrates. eLife.

[bib50] Crawford JE, Amaru R, Song J, Julian CG, Racimo F, Cheng JY, Guo X, Yao J, Ambale-Venkatesh B, Lima JA, Rotter JI, Stehlik J, Moore LG, Prchal JT, Nielsen R (2017). Natural selection on genes related to cardiovascular health in high-altitude adapted andeans. American Journal of Human Genetics.

[bib51] Daff S (2010). NO synthase: structures and mechanisms. Nitric Oxide.

[bib52] Danielson PB (2002). The cytochrome P450 superfamily: biochemistry, evolution and drug metabolism in humans. Current Drug Metabolism.

[bib53] Dao JH, Kurzeja RJM, Morachis JM, Veith H, Lewis J, Yu V, Tegley CM, Tagari P (2009). Kinetic characterization and identification of a novel inhibitor of hypoxia-inducible factor prolyl hydroxylase 2 using a time-resolved fluorescence resonance energy transfer-based assay technology. Analytical Biochemistry.

[bib54] Daubner SC, Le T, Wang S (2011). Tyrosine hydroxylase and regulation of dopamine synthesis. Archives of Biochemistry and Biophysics.

[bib55] Dawson VL, Dawson TM (1996). Nitric oxide neurotoxicity. Journal of Chemical Neuroanatomy.

[bib56] Decker A, Solomon EI (2005). Dioxygen activation by copper, heme and non-heme iron enzymes: comparison of electronic structures and reactivities. Current Opinion in Chemical Biology.

[bib57] Deschoemaeker S, Di Conza G, Lilla S, Martín-Pérez R, Mennerich D, Boon L, Hendrikx S, Maddocks ODK, Marx C, Radhakrishnan P, Prenen H, Schneider M, Myllyharju J, Kietzmann T, Vousden KH, Zanivan S, Mazzone M (2015). PHD1 regulates p53-mediated colorectal cancer chemoresistance. EMBO Molecular Medicine.

[bib58] Dhillon S (2019). Roxadustat: First Global Approval. Drugs.

[bib59] Dhillon S (2020). Daprodustat: First Approval. Drugs.

[bib60] Dobrynin G, McAllister TE, Leszczynska KB, Ramachandran S, Krieg AJ, Kawamura A, Hammond EM (2017). KDM4A regulates HIF-1 levels through H3K9me3. Scientific Reports.

[bib61] Donkó Á, Péterfi Z, Sum A, Leto T, Geiszt M (2005). Dual oxidases. Philosophical Transactions of the Royal Society B.

[bib62] Donovan L, Welford SM, Haaga J, LaManna J, Strohl KP (2010). Hypoxia--implications for pharmaceutical developments. Sleep & Breathing = Schlaf & Atmung.

[bib63] Duranteau J, Chandel NS, Kulisz A, Shao Z, Schumacker PT (1998). Intracellular signaling by reactive oxygen species during hypoxia in cardiomyocytes. Journal of Biological Chemistry.

[bib64] Efimov I, Basran J, Thackray SJ, Handa S, Mowat CG, Raven EL (2011). Structure and reaction mechanism in the heme dioxygenases. Biochemistry.

[bib65] Ehrismann D, Flashman E, Genn DN, Mathioudakis N, Hewitson KS, Ratcliffe PJ, Schofield CJ (2007). Studies on the activity of the hypoxia-inducible-factor hydroxylases using an oxygen consumption assay. The Biochemical Journal.

[bib66] Eichstaedt CA, Antão T, Pagani L, Cardona A, Kivisild T, Mormina M (2014). The Andean adaptive toolkit to counteract high altitude maladaptation: genome-wide and phenotypic analysis of the Collas. PLOS ONE.

[bib67] Elayan IM, Axley MJ, Prasad PV, Ahlers ST, Auker CR (2000). Effect of hyperbaric oxygen treatment on nitric oxide and oxygen free radicals in rat brain. Journal of Neurophysiology.

[bib68] Elbers F, Woite C, Antoni V, Stein S, Funakoshi H, Nakamura T, Schares G, Däubener W, Eller SK (2016). Negative impact of hypoxia on tryptophan 2,3-Dioxygenase Function. Mediators of Inflammation.

[bib69] Emerling BM, Platanias LC, Black E, Nebreda AR, Davis RJ, Chandel NS (2005). Mitochondrial reactive oxygen species activation of p38 mitogen-activated protein kinase is required for hypoxia signaling. Molecular and Cellular Biology.

[bib70] Emerling BM, Weinberg F, Snyder C, Burgess Z, Mutlu GM, Viollet B, Budinger GRS, Chandel NS (2009). Hypoxic activation of AMPK is dependent on mitochondrial ROS but independent of an increase in AMP/ATP ratio. Free Radical Biology & Medicine.

[bib71] Epstein AC, Gleadle JM, McNeill LA, Hewitson KS, O’Rourke J, Mole DR, Mukherji M, Metzen E, Wilson MI, Dhanda A, Tian YM, Masson N, Hamilton DL, Jaakkola P, Barstead R, Hodgkin J, Maxwell PH, Pugh CW, Schofield CJ, Ratcliffe PJ (2001). *C. elegans* EGL-9 and mammalian homologs define a family of dioxygenases that regulate HIF by prolyl hydroxylation. Cell.

[bib72] Ferguson-Miller S, Babcock GT (1996). Heme/copper terminal oxidases. Chemical Reviews.

[bib73] Finney J, Moon HJ, Ronnebaum T, Lantz M, Mure M (2014). Human copper-dependent amine oxidases. Archives of Biochemistry and Biophysics.

[bib74] Fletcher SC, Coleman ML (2020). Human 2-oxoglutarate-dependent oxygenases: nutrient sensors, stress responders, and disease mediators. Biochemical Society Transactions.

[bib75] Fong G-H, Takeda K (2008). Role and regulation of prolyl hydroxylase domain proteins. Cell Death & Differentiation.

[bib76] Fradette C, Du Souich P (2004). Effect of hypoxia on cytochrome P450 activity and expression. Current Drug Metabolism.

[bib77] Freedman SJ, Sun ZYJ, Poy F, Kung AL, Livingston DM, Wagner G, Eck MJ (2002). Structural basis for recruitment of CBP/p300 by hypoxia-inducible factor-1 alpha. PNAS.

[bib78] Fuhrmann DC, Brüne B (2017). Mitochondrial composition and function under the control of hypoxia. Redox Biology.

[bib79] Gardie B, Percy MJ, Hoogewijs D, Chowdhury R, Bento C, Arsenault PR, Richard S, Almeida H, Ewing J, Lambert F, McMullin MF, Schofield CJ, Lee FS (2014). The role of PHD2 mutations in the pathogenesis of erythrocytosis. Hypoxia.

[bib80] Ge W, Wolf A, Feng T, Ho C-H, Sekirnik R, Zayer A, Granatino N, Cockman ME, Loenarz C, Loik ND, Hardy AP, Claridge TDW, Hamed RB, Chowdhury R, Gong L, Robinson CV, Trudgian DC, Jiang M, Mackeen MM, Mccullagh JS, Gordiyenko Y, Thalhammer A, Yamamoto A, Yang M, Liu-Yi P, Zhang Z, Schmidt-Zachmann M, Kessler BM, Ratcliffe PJ, Preston GM, Coleman ML, Schofield CJ (2012). Oxygenase-catalyzed ribosome hydroxylation occurs in prokaryotes and humans. Nature Chemical Biology.

[bib81] Gnaiger E, Steinlechner-Maran R, Méndez G, Eberl T, Margreiter R (1995). Control of mitochondrial and cellular respiration by oxygen. Journal of Bioenergetics and Biomembranes.

[bib82] Green CD, Olson LK (2011). Modulation of palmitate-induced endoplasmic reticulum stress and apoptosis in pancreatic β-cells by stearoyl-CoA desaturase and Elovl6. American Journal of Physiology. Endocrinology and Metabolism.

[bib83] Guengerich FP (2007). Mechanisms of cytochrome P450 substrate oxidation: MiniReview. Journal of Biochemical and Molecular Toxicology.

[bib84] Guo J, Chakraborty AA, Liu P, Gan W, Zheng X, Inuzuka H, Wang B, Zhang J, Zhang L, Yuan M, Novak J, Cheng JQ, Toker A, Signoretti S, Zhang Q, Asara JM, Kaelin WG, Wei W (2016). pVHL suppresses kinase activity of Akt in a proline-hydroxylation-dependent manner. Science.

[bib85] Gupta N, Wish JB (2017). Hypoxia-Inducible factor prolyl hydroxylase inhibitors: A potential new treatment for anemia in patients with CKD. American Journal of Kidney Diseases.

[bib86] Guzy RD, Mack MM, Schumacker PT (2007). Mitochondrial complex III is required for hypoxia-induced ROS production and gene transcription in yeast. Antioxidants & Redox Signaling.

[bib87] Haase VH (2013). Regulation of erythropoiesis by hypoxia-inducible factors. Blood Reviews.

[bib88] Hancock RL, Dunne K, Walport LJ, Flashman E, Kawamura A (2015). Epigenetic regulation by histone demethylases in hypoxia. Epigenomics.

[bib89] Hancock RL, Masson N, Dunne K, Flashman E, Kawamura A (2017). The activity of JmjC Histone lysine demethylase KDM4A is Highly sensitive to oxygen concentrations. ACS Chemical Biology.

[bib90] Hatefi Y (1985). The mitochondrial electron transport and oxidative phosphorylation system. Annual Review of Biochemistry.

[bib91] Hernansanz-Agustín P, Ramos E, Navarro E, Parada E, Sánchez-López N, Peláez-Aguado L, Cabrera-García JD, Tello D, Buendia I, Marina A, Egea J, López MG, Bogdanova A, Martínez-Ruiz A (2017). Mitochondrial complex I deactivation is related to superoxide production in acute hypoxia. Redox Biology.

[bib92] Hess D, Chisholm JW, Igal RA (2010). Inhibition of stearoylCoA desaturase activity blocks cell cycle progression and induces programmed cell death in lung cancer cells. PLOS ONE.

[bib93] Hillringhaus L, Yue WW, Rose NR, Ng SS, Gileadi C, Loenarz C, Bello SH, Bray JE, Schofield CJ, Oppermann U (2011). Structural and evolutionary basis for the dual substrate selectivity of human KDM4 histone demethylase family. The Journal of Biological Chemistry.

[bib94] Hirsilä M, Koivunen P, Günzler V, Kivirikko KI, Myllyharju J (2003). Characterization of the human prolyl 4-Hydroxylases that modify the hypoxia-inducible factor. Journal of Biological Chemistry.

[bib95] Ho JJD, Man HSJ, Marsden PA (2012). Nitric oxide signaling in hypoxia. Journal of Molecular Medicine.

[bib96] Holdsworth MJ, Gibbs DJ (2020). Comparative biology of oxygen sensing in plants and animals. Current Biology.

[bib97] Hölscher M, Silter M, Krull S, von Ahlen M, Hesse A, Schwartz P, Wielockx B, Breier G, Katschinski DM, Zieseniss A (2011). Cardiomyocyte-specific prolyl-4-hydroxylase domain 2 knock out protects from acute myocardial ischemic injury. The Journal of Biological Chemistry.

[bib98] Hong S, Cho YW, Yu LR, Yu H, Veenstra TD, Ge K (2007). Identification of JmjC domain-containing UTX and JMJD3 as histone H3 lysine 27 demethylases. PNAS.

[bib99] Huang X, Groves JT (2018). Oxygen activation and radical transformations in heme proteins and metalloporphyrins. Chemical Reviews.

[bib100] Hughes AL, Todd BL, Espenshade PJ (2005). SREBP Pathway responds to sterols and functions as an oxygen sensor in fission yeast. Cell.

[bib101] Hughes BT, Espenshade PJ (2008). Oxygen-regulated degradation of fission yeast SREBP by Ofd1, a prolyl hydroxylase family member. The EMBO Journal.

[bib102] Hui AS, Striet JB, Gudelsky G, Soukhova GK, Gozal E, Beitner-Johnson D, Guo SZ, Sachleben LR, Haycock JW, Gozal D, Czyzyk-Krzeska MF (2003). Regulation of catecholamines by sustained and intermittent hypoxia in neuroendocrine cells and sympathetic neurons. Hypertension.

[bib103] Islam MS, Leissing TM, Chowdhury R, Hopkinson RJ, Schofield CJ (2018). 2-Oxoglutarate-Dependent Oxygenases. Annual Review of Biochemistry.

[bib104] Itoh S (2006). Mononuclear copper active-oxygen complexes. Current Opinion in Chemical Biology.

[bib105] Iturriaga R, Alcayaga J (2004). Neurotransmission in the carotid body: transmitters and modulators between glomus cells and petrosal ganglion nerve terminals. Brain Research. Brain Research Reviews.

[bib106] Iturriaga R, Alcayaga J, Gonzalez C, Gonzalez C, Nurse CA, Peers C (2009). Arterial Chemoreceptors.

[bib107] Ivan M, Kaelin WG (2017). The EGLN-HIF O 2 -Sensing system: Multiple Inputs and feedbacks. Molecular Cell.

[bib108] Ivanov I, Saam J, Kuhn H, Holzhütter HG (2005). Dual role of oxygen during lipoxygenase reactions. The FEBS Journal.

[bib109] Ivanov I, Heydeck D, Hofheinz K, Roffeis J, O’Donnell VB, Kuhn H, Walther M (2010). Molecular enzymology of lipoxygenases. Archives of Biochemistry and Biophysics.

[bib110] Ivanov I, Kuhn H, Heydeck D (2015). Structural and functional biology of arachidonic acid 15-lipoxygenase-1 (ALOX15). Gene.

[bib111] Iwase S, Lan F, Bayliss P, de la Torre-Ubieta L, Huarte M, Qi HH, Whetstine JR, Bonni A, Roberts TM, Shi Y (2007). The X-linked mental retardation gene SMCX/JARID1C defines a family of histone H3 lysine 4 demethylases. Cell.

[bib112] Jabłońska J, Tawfik DS (2021). The evolution of oxygen-utilizing enzymes suggests early biosphere oxygenation. Nature Ecology & Evolution.

[bib113] Jacovas VC, Couto-Silva CM, Nunes K, Lemes RB, de Oliveira MZ, Salzano FM, Bortolini MC, Hünemeier T (2018). Selection scan reveals three new loci related to high altitude adaptation in native andeans. Scientific Reports.

[bib114] Jagannathan L, Cuddapah S, Costa M (2016). Oxidative stress under ambient and physiological oxygen tension in tissue culture. Current Pharmacology Reports.

[bib115] Jasniewski AJ, Que L (2018). DIoxygen activation by nonheme diiron enzymes: diverse dioxygen adducts, high-valent intermediates, and related model complexes. Chemical Reviews.

[bib116] Jia G, Fu Y, Zhao X, Dai Q, Zheng G, Yang Y, Yi C, Lindahl T, Pan T, Yang Y-G, He C (2011). N6-methyladenosine in nuclear RNA is a major substrate of the obesity-associated FTO. Nature Chemical Biology.

[bib117] Johansson C, Tumber A, Che K, Cain P, Nowak R, Gileadi C, Oppermann U (2014). The roles of Jumonji-type oxygenases in human disease. Epigenomics.

[bib118] Jones DP (1981). Hypoxia and drug metabolism. Biochemical Pharmacology.

[bib119] Juránek I, Suzuki H, Yamamoto S (1999). Affinities of various mammalian arachidonate lipoxygenases and cyclooxygenases for molecular oxygen as substrate. Biochimica et Biophysica Acta (BBA) - Molecular and Cell Biology of Lipids.

[bib120] Kaelin WG, Ratcliffe PJ (2008). Oxygen sensing by metazoans: the central role of the HIF hydroxylase pathway. Molecular Cell.

[bib121] Kamphorst JJ, Cross JR, Fan J, de Stanchina E, Mathew R, White EP, Thompson CB, Rabinowitz JD (2013). Hypoxic and Ras-transformed cells support growth by scavenging unsaturated fatty acids from lysophospholipids. PNAS.

[bib122] Katz IR (1980). Oxygen affinity of tyrosine and tryptophan hydroxylases in synaptosomes. Journal of Neurochemistry.

[bib123] Kim J-H, Choi TG, Park S, Yun HR, Nguyen NNY, Jo YH, Jang M, Kim J, Kim J, Kang I, Ha J, Murphy MP, Tang DG, Kim SS (2018). Mitochondrial ROS-derived PTEN oxidation activates PI3K pathway for mTOR-induced myogenic autophagy. Cell Death and Differentiation.

[bib124] Köditz J, Nesper J, Wottawa M, Stiehl DP, Camenisch G, Franke C, Myllyharju J, Wenger RH, Katschinski DM (2007). Oxygen-dependent ATF-4 stability is mediated by the PHD3 oxygen sensor. Blood.

[bib125] Koivunen P, Hirsilä M, Günzler V, Kivirikko KI, Myllyharju J (2004). Catalytic properties of the asparaginyl hydroxylase (FIH) in the oxygen sensing pathway are distinct from those of its prolyl 4-hydroxylases. The Journal of Biological Chemistry.

[bib126] Kolawole AO, Hixon BP, Dameron LS, Chrisman IM, Smirnov VV (2015). Catalytic activity of human indoleamine 2,3-dioxygenase (hIDO1) at low oxygen. Archives of Biochemistry and Biophysics.

[bib127] Kooistra SM, Helin K (2012). Molecular mechanisms and potential functions of histone demethylases. Nature Reviews. Molecular Cell Biology.

[bib128] Koury MJ, Haase VH (2015). Anaemia in kidney disease: harnessing hypoxia responses for therapy. Nature Reviews. Nephrology.

[bib129] Kuhn H, Banthiya S, van Leyen K (2015). Mammalian lipoxygenases and their biological relevance. Biochimica et Biophysica Acta (BBA) - Molecular and Cell Biology of Lipids.

[bib130] Kulisz A, Chen N, Chandel NS, Shao Z, Schumacker PT (2002). Mitochondrial ROS initiate phosphorylation of p38 MAP kinase during hypoxia in cardiomyocytes. American Journal of Physiology. Lung Cellular and Molecular Physiology.

[bib131] Kumar GK, Kim DK, Lee MS, Ramachandran R, Prabhakar NR (2003). Activation of tyrosine hydroxylase by intermittent hypoxia: involvement of serine phosphorylation. Journal of Applied Physiology.

[bib132] Kunze R, Zhou W, Veltkamp R, Wielockx B, Breier G, Marti HH (2012). Neuron-specific prolyl-4-hydroxylase domain 2 knockout reduces brain injury after transient cerebral ischemia. Stroke.

[bib133] Lando D, Peet DJ, Gorman JJ, Whelan DA, Whitelaw ML, Bruick RK (2002a). FIH-1 is an asparaginyl hydroxylase enzyme that regulates the transcriptional activity of hypoxia-inducible factor. Genes & Development.

[bib134] Lando D, Peet DJ, Whelan DA, Gorman JJ, Whitelaw ML (2002b). Asparagine hydroxylation of the HIF transactivation domain a hypoxic switch. Science.

[bib135] Laukka T, Mariani CJ, Ihantola T, Cao JZ, Hokkanen J, Kaelin WG, Godley LA, Koivunen P (2016). Fumarate and Succinate Regulate Expression of Hypoxia-inducible Genes via TET Enzymes. The Journal of Biological Chemistry.

[bib136] Lecomte JTJ, Vuletich DA, Lesk AM (2005). Structural divergence and distant relationships in proteins: evolution of the globins. Current Opinion in Structural Biology.

[bib137] Lee JW, Ko J, Ju C, Eltzschig HK (2019). Hypoxia signaling in human diseases and therapeutic targets. Experimental & Molecular Medicine.

[bib138] Lewis EA, Tolman WB (2004). Reactivity of dioxygen-copper systems. Chemical Reviews.

[bib139] Li F, Sonveaux P, Rabbani ZN, Liu S, Yan B, Huang Q, Vujaskovic Z, Dewhirst MW, Li C-Y (2007). Regulation of HIF-1alpha stability through S-nitrosylation. Molecular Cell.

[bib140] Li J, Zhang T, Ren T, Liao X, Hao Y, Lim JS, Lee J-H, Li M, Shao J, Liu R (2022). Oxygen-sensitive methylation of ULK1 is required for hypoxia-induced autophagy. Nature Communications.

[bib141] Liu X, Wang J, Boyer JA, Gong W, Zhao S, Xie L, Wu Q, Zhang C, Jain K, Guo Y, Rodriguez J, Li M, Uryu H, Liao C, Hu L, Zhou J, Shi X, Tsai Y-H, Yan Q, Luo W, Chen X, Strahl BD, von Kriegsheim A, Zhang Q, Wang GG, Baldwin AS, Zhang Q (2022). Histone H3 proline 16 hydroxylation regulates mammalian gene expression. Nature Genetics.

[bib142] López-Barneo J, Ortega-Sáenz P, Pardal R, Pascual A, Piruat JI (2008). Carotid body oxygen sensing. The European Respiratory Journal.

[bib143] Lorenzo FR, Huff C, Myllymäki M, Olenchock B, Swierczek S, Tashi T, Gordeuk V, Wuren T, Ri-Li G, McClain DA, Khan TM, Koul PA, Guchhait P, Salama ME, Xing J, Semenza GL, Liberzon E, Wilson A, Simonson TS, Jorde LB, Kaelin WG, Koivunen P, Prchal JT (2014). A genetic mechanism for tibetan high-altitude adaptation. Nature Genetics.

[bib144] Mahon PC, Hirota K, Semenza GL (2001). FIH-1: a novel protein that interacts with HIF-1α and VHL to mediate repression of HIF-1 transcriptional activity. Genes & Development.

[bib145] Majmundar AJ, Wong WJ, Simon MC (2010). Hypoxia-inducible factors and the response to hypoxic stress. Molecular Cell.

[bib146] Markolovic S, Wilkins SE, Schofield CJ (2015). Protein hydroxylation catalyzed by 2-oxoglutarate-dependent oxygenases. The Journal of Biological Chemistry.

[bib147] Martin DS, Khosravi M, Grocott MP, Mythen MG (2010). Concepts in hypoxia reborn. Critical Care.

[bib148] Martinez S, Hausinger RP (2015). Catalytic mechanisms of fe(ii)- and 2-oxoglutarate-dependent oxygenases. The Journal of Biological Chemistry.

[bib149] Mas-Bargues C, Sanz-Ros J, Román-Domínguez A, Inglés M, Gimeno-Mallench L, El Alami M, Viña-Almunia J, Gambini J, Viña J, Borrás C (2019). Relevance of oxygen concentration in stem cell culture for regenerative medicine. International Journal of Molecular Sciences.

[bib150] Mashima R, Okuyama T (2015). The role of lipoxygenases in pathophysiology; new insights and future perspectives. Redox Biology.

[bib151] Massey V (2002). The reactivity of oxygen with flavoproteins. International Congress Series.

[bib152] Masson N, Keeley TP, Giuntoli B, White MD, Puerta ML, Perata P, Hopkinson RJ, Flashman E, Licausi F, Ratcliffe PJ (2019). Conserved N-terminal cysteine dioxygenases transduce responses to hypoxia in animals and plants. Science.

[bib153] Mauer J, Luo X, Blanjoie A, Jiao X, Grozhik AV, Patil DP, Linder B, Pickering BF, Vasseur J-J, Chen Q, Gross SS, Elemento O, Debart F, Kiledjian M, Jaffrey SR (2017). Reversible methylation of m6Am in the 5′ cap controls mRNA stability. Nature.

[bib154] McIntyre NR, Lowe EW, Belof JL, Ivkovic M, Shafer J, Space B, Merkler DJ (2010). Evidence for substrate preorganization in the peptidylglycine α-amidating monooxygenase reaction describing the contribution of ground state structure to hydrogen tunneling. Journal of the American Chemical Society.

[bib155] McKeown SR (2014). Defining normoxia, physoxia and hypoxia in tumours-implications for treatment response. The British Journal of Radiology.

[bib156] Merkler DJ (1994). C-terminal amidated peptides: production by the in vitro enzymatic amidation of glycine-extended peptides and the importance of the amide to bioactivity. Enzyme and Microbial Technology.

[bib157] Migita CT, Matera KM, Ikeda-Saito M, Olson JS, Fujii H, Yoshimura T, Zhou H, Yoshida T (1998). The oxygen and carbon monoxide reactions of heme oxygenase. The Journal of Biological Chemistry.

[bib158] Minamishima YA, Moslehi J, Bardeesy N, Cullen D, Bronson RT, Kaelin WG (2008). Somatic inactivation of the PHD2 prolyl hydroxylase causes polycythemia and congestive heart failure. Blood.

[bib159] Moncada S, Higgs EA (1991). Endogenous nitric oxide: physiology, pathology and clinical relevance. European Journal of Clinical Investigation.

[bib160] Monson EK, Ditta GS, Helinski DR (1995). THe oxygen sensor protein, fixl, of rhizobium meliloti. Journal OF Biological Chemistry.

[bib161] Morris NJ, Ducret A, Aebersold R, Ross SA, Keller SR, Lienhard GE (1997). Membrane amine oxidase cloning and identification as a major protein in the adipocyte plasma membrane. The Journal of Biological Chemistry.

[bib162] Moser SC, Bensaddek D, Ortmann B, Maure J-F, Mudie S, Blow JJ, Lamond AI, Swedlow JR, Rocha S (2013). PHD1 Links cell-cycle progression to oxygen sensing through hydroxylation of the centrosomal protein cep192. Developmental Cell.

[bib163] Namslauer A, Brzezinski P (2004). Structural elements involved in electron-coupled proton transfer in cytochrome c oxidase. FEBS Letters.

[bib164] Nangaku M, Eckardt KU (2007). Hypoxia and the HIF system in kidney disease. Journal of Molecular Medicine.

[bib165] Nolfi-Donegan D, Braganza A, Shiva S (2020). Mitochondrial electron transport chain: Oxidative phosphorylation, oxidant production, and methods of measurement. Redox Biology.

[bib166] Ortiz-Prado E, Dunn JF, Vasconez J, Castillo D, Viscor G (2019). Partial pressure of oxygen in the human body: a general review. American Journal of Blood Research.

[bib167] Palfey BA, McDonald CA (2010). Control of catalysis in flavin-dependent monooxygenases. Archives of Biochemistry and Biophysics.

[bib168] Palmer LA, Doctor A, Chhabra P, Sheram ML, Laubach VE, Karlinsey MZ, Forbes MS, Macdonald T, Gaston B (2007). S-nitrosothiols signal hypoxia-mimetic vascular pathology. The Journal of Clinical Investigation.

[bib169] Parati G, Agostoni P, Basnyat B, Bilo G, Brugger H, Coca A, Festi L, Giardini G, Lironcurti A, Luks AM, Maggiorini M, Modesti PA, Swenson ER, Williams B, Bärtsch P, Torlasco C (2018). Clinical recommendations for high altitude exposure of individuals with pre-existing cardiovascular conditions: A joint statement by the European Society of Cardiology, the Council on Hypertension of the European Society of Cardiology, the European Society of Hypertension, the International Society of Mountain Medicine, the Italian Society of Hypertension and the Italian Society of Mountain Medicine. European Heart Journal.

[bib170] Paton CM, Ntambi JM (2009). Biochemical and physiological function of stearoyl-CoA desaturase. American Journal of Physiology. Endocrinology and Metabolism.

[bib171] Peng Y, Yang Z, Zhang H, Cui C, Qi X, Luo X, Tao X, Wu T, Chen H, Shi H, Su B (2011). Genetic variations in Tibetan populations and high-altitude adaptation at the Himalayas. Molecular Biology and Evolution.

[bib172] Percy MJ, Zhao Q, Flores A, Harrison C, Lappin TRJ, Maxwell PH, McMullin MF, Lee FS (2006). A family with erythrocytosis establishes A role for prolyl hydroxylase domain protein 2 in oxygen homeostasis. PNAS.

[bib173] Petersen LC, Nicholls P, Degn H (1974). The effect of energization on the apparent Michaelis-Mentne constant for oxygen in mitochondrial respiration. The Biochemical Journal.

[bib174] Pfeffer MA, Burdmann EA, Chen C-Y, Cooper ME, de Zeeuw D, Eckardt K-U, Feyzi JM, Ivanovich P, Kewalramani R, Levey AS, Lewis EF, McGill JB, McMurray JJV, Parfrey P, Parving H-H, Remuzzi G, Singh AK, Solomon SD, Toto R (2009). A trial of darbepoetin alfa in type 2 diabetes and chronic kidney disease. New England Journal of Medicine.

[bib175] Place TL, Domann FE, Case AJ (2017). Limitations of oxygen delivery to cells in culture: An underappreciated problem in basic and translational research. Free Radical Biology & Medicine.

[bib176] Ponnaluri VKC, Maciejewski JP, Mukherji M (2013). A mechanistic overview of TET-mediated 5-methylcytosine oxidation. Biochemical and Biophysical Research Communications.

[bib177] Portolés J, Martín L, Broseta JJ, Cases A (2021). Anemia in chronic kidney disease: From pathophysiology and current treatments, to future agents. Frontiers in Medicine.

[bib178] Poulos TL, Johnson EF, Ortiz de Montellano PR (2005). Cytochrome P450: Structure, Mechanism, and Biochemistry.

[bib179] Prigge ST, Eipper BA, Mains RE, Amzel LM (2004). Dioxygen Binds End-On to Mononuclear Copper in a Precatalytic Enzyme Complex. Science.

[bib180] Qi X, Cui C, Peng Y, Zhang X, Yang Z, Zhong H, Zhang H, Xiang K, Cao X, Wang Y, Wu T, Chen H, Shi H, Su B (2013). Genetic evidence of paleolithic colonization and neolithic expansion of modern humans on the tibetan plateau. Molecular Biology and Evolution.

[bib181] Qian X, Li X, Shi Z, Bai X, Xia Y, Zheng Y, Xu D, Chen F, You Y, Fang J, Hu Z, Zhou Q, Lu Z (2019). KDM3A Senses Oxygen Availability to Regulate PGC-1α-Mediated Mitochondrial Biogenesis. Molecular Cell.

[bib182] Raghuraman G, Prabhakar NR, Kumar GK (2012). Differential regulation of tyrosine hydroxylase by continuous and intermittent hypoxia. Arterial Chemoreception.

[bib183] Ragsdale SW, Yi L (2011). Thiol/Disulfide Redox Switches in the Regulation of Heme Binding to Proteins. Antioxidants & Redox Signaling.

[bib184] Raven EL (2017). A short history of heme dioxygenases: rise, fall and rise again. Journal of Biological Inorganic Chemistry.

[bib185] Repessé X, Moldes M, Muscat A, Vatier C, Chetrite G, Gille T, Planes C, Filip A, Mercier N, Duranteau J, Fève B (2015). Hypoxia inhibits semicarbazide-sensitive amine oxidase activity in adipocytes. Molecular and Cellular Endocrinology.

[bib186] Roach RC, Hackett PH (2001). Frontiers of hypoxia research: acute mountain sickness. Journal of Experimental Biology.

[bib187] Roberts KM, Fitzpatrick PF (2013). Mechanisms of tryptophan and tyrosine hydroxylase. IUBMB Life.

[bib188] Robinson MA, Baumgardner JE, Otto CM (2011). Oxygen-dependent regulation of nitric oxide production by inducible nitric oxide synthase. Free Radical Biology & Medicine.

[bib189] Rodriguez J, Herrero A, Li S, Rauch N, Quintanilla A, Wynne K, Krstic A, Acosta JC, Taylor C, Schlisio S, von Kriegsheim A (2018). PHD3 Regulates p53 Protein Stability by Hydroxylating Proline 359. Cell Reports.

[bib190] Romero E, Gómez Castellanos JR, Gadda G, Fraaije MW, Mattevi A (2018). Same Substrate, Many Reactions: Oxygen Activation in Flavoenzymes. Chemical Reviews.

[bib191] Rose NR, McDonough MA, King ONF, Kawamura A, Schofield CJ (2011). Inhibition of 2-oxoglutarate dependent oxygenases. Chemical Society Reviews.

[bib192] Rostrup M, Fossbakk A, Hauge A, Kleppe R, Gnaiger E, Haavik J (2008). Oxygen dependence of tyrosine hydroxylase. Amino Acids.

[bib193] Santolini J, Adak S, Curran CM, Stuehr DJ (2001a). A kinetic simulation model that describes catalysis and regulation in nitric-oxide synthase. The Journal of Biological Chemistry.

[bib194] Santolini J, Meade AL, Stuehr DJ (2001b). Differences in three kinetic parameters underpin the unique catalytic profiles of nitric-oxide synthases I, II, and III. The Journal of Biological Chemistry.

[bib195] Scandurra FM, Gnaiger E (2010). Cell respiration under hypoxia: facts and artefacts in mitochondrial oxygen kinetics. Oxygen Transport to Tissue.

[bib196] Scarpulla RC, Vega RB, Kelly DP (2012). Transcriptional integration of mitochondrial biogenesis. Trends in Endocrinology & Metabolism.

[bib197] Scheinfeldt LB, Soi S, Thompson S, Ranciaro A, Woldemeskel D, Beggs W, Lambert C, Jarvis JP, Abate D, Belay G, Tishkoff SA (2012). Genetic adaptation to high altitude in the Ethiopian highlands. Genome Biology.

[bib198] Schmidt SK, Ebel S, Keil E, Woite C, Ernst JF, Benzin AE, Rupp J, Däubener W (2013). Regulation of IDO activity by oxygen supply: inhibitory effects on antimicrobial and immunoregulatory functions. PLOS ONE.

[bib199] Schnell PO, Ignacak ML, Bauer AL, Striet JB, Paulding WR, Czyzyk-Krzeska MF (2003). Regulation of tyrosine hydroxylase promoter activity by the von Hippel-Lindau tumor suppressor protein and hypoxia-inducible transcription factors. Journal of Neurochemistry.

[bib200] Schofield CJ, Ratcliffe PJ (2004). Oxygen sensing by HIF hydroxylases. Nature Reviews. Molecular Cell Biology.

[bib201] Scholz CC, Rodriguez J, Pickel C, Burr S, Fabrizio JA, Nolan KA, Spielmann P, Cavadas MAS, Crifo B, Halligan DN, Nathan JA, Peet DJ, Wenger RH, Von Kriegsheim A, Cummins EP, Taylor CT (2016). FIH Regulates cellular metabolism through hydroxylation of the deubiquitinase OTUB1. PLOS Biology.

[bib202] Segura I, Lange C, Knevels E, Moskalyuk A, Pulizzi R, Eelen G, Chaze T, Tudor C, Boulegue C, Holt M, Daelemans D, Matondo M, Ghesquière B, Giugliano M, Ruiz de Almodovar C, Dewerchin M, Carmeliet P (2016). The oxygen sensor PHD2 Controls dendritic spines and synapses via modification of filamin A. Cell Reports.

[bib203] Semenza GL (2005). New insights into nNOS regulation of vascular homeostasis. Journal of Clinical Investigation.

[bib204] Shen SH, Wertz DL, Klinman JP (2012). Implication for functions of the ectopic adipocyte copper amine oxidase (AOC3) from purified enzyme and cell-based kinetic studies. PLOS ONE.

[bib205] Shmakova A, Batie M, Druker J, Rocha S (2014). Chromatin and oxygen sensing in the context of JmjC histone demethylases. The Biochemical Journal.

[bib206] Sim J, Cowburn AS, Palazon A, Madhu B, Tyrakis PA, Macías D, Bargiela DM, Pietsch S, Gralla M, Evans CE, Kittipassorn T, Chey YCJ, Branco CM, Rundqvist H, Peet DJ, Johnson RS (2018). The factor inhibiting HIF Asparaginyl hydroxylase regulates oxidative metabolism and accelerates metabolic adaptation to hypoxia. Cell Metabolism.

[bib207] Simonson TS, Yang Y, Huff CD, Yun H, Qin G, Witherspoon DJ, Bai Z, Lorenzo FR, Xing J, Jorde LB, Prchal JT, Ge R (2010). Genetic evidence for high-altitude adaptation in tibet. Science.

[bib208] Simpson PD, Eipper BA, Katz MJ, Gandara L, Wappner P, Fischer R, Hodson EJ, Ratcliffe PJ, Masson N (2015). Striking oxygen sensitivity of the peptidylglycine α-amidating monooxygenase (pam) in neuroendocrine cells. The Journal of Biological Chemistry.

[bib209] Singh AK, Szczech L, Tang KL, Barnhart H, Sapp S, Wolfson M, Reddan D (2006). Correction of anemia with epoetin alfa in chronic kidney disease. New England Journal of Medicine.

[bib210] Singleton RS, Liu-Yi P, Formenti F, Ge W, Sekirnik R, Fischer R, Adam J, Pollard PJ, Wolf A, Thalhammer A, Loenarz C, Flashman E, Yamamoto A, Coleman ML, Kessler BM, Wappner P, Schofield CJ, Ratcliffe PJ, Cockman ME (2014). OGFOD1 catalyzes prolyl hydroxylation of RPS23 and is involved in translation control and stress granule formation. PNAS.

[bib211] Solomon EI, Chen P, Metz M, Lee SK, Palmer AE (2001). Oxygen binding, activation, and reduction to water by copper proteins. Angewandte Chemie.

[bib212] Sono M, Roach MP, Coulter ED, Dawson JH (1996). Heme-containing oxygenases. Chemical Reviews.

[bib213] Souvannakitti D, Kumar GK, Fox A, Prabhakar NR (2009). Neonatal intermittent hypoxia leads to long-lasting facilitation of acute hypoxia-evoked catecholamine secretion from rat chromaffin cells. Journal of Neurophysiology.

[bib214] Strowitzki MJ, Cummins EP, Taylor CT (2019). Protein hydroxylation by hypoxia-inducible factor (HIF) Hydroxylases: Unique or ubiquitous?. Cells.

[bib215] Stuehr DJ, Santolini J, Wang ZQ, Wei CC, Adak S (2004). Update on mechanism and catalytic regulation in the NO synthases. The Journal of Biological Chemistry.

[bib216] Takeda K, Ho VC, Takeda H, Duan L-J, Nagy A, Fong G-H (2006). Placental but not heart defects are associated with elevated hypoxia-inducible factor alpha levels in mice lacking prolyl hydroxylase domain protein 2. Molecular and Cellular Biology.

[bib217] Tarhonskaya H, Chowdhury R, Leung IKH, Loik ND, McCullagh JSO, Claridge TDW, Schofield CJ, Flashman E (2014). Investigating the contribution of the active site environment to the slow reaction of hypoxia-inducible factor prolyl hydroxylase domain 2 with oxygen. The Biochemical Journal.

[bib218] Taylor CT, McElwain JC (2010). Ancient atmospheres and the evolution of oxygen sensing via the hypoxia-inducible factor in metazoans. Physiology.

[bib219] Thackray SJ, Bruckmann C, Mowat CG, Forouhar F, Chapman SK, Tong L, Scott RA (2011). Encyclopedia of Inorganic and Bioinorganic Chemistry.

[bib220] Thienpont B, Steinbacher J, Zhao H, D’Anna F, Kuchnio A, Ploumakis A, Ghesquière B, Van Dyck L, Boeckx B, Schoonjans L, Hermans E, Amant F, Kristensen VN, Peng Koh K, Mazzone M, Coleman M, Carell T, Carmeliet P, Lambrechts D (2016). Tumour hypoxia causes DNA hypermethylation by reducing TET activity. Nature.

[bib221] Tian Y-M, Yeoh KK, Lee MK, Eriksson T, Kessler BM, Kramer HB, Edelmann MJ, Willam C, Pugh CW, Schofield CJ, Ratcliffe PJ (2011). Differential sensitivity of hypoxia inducible factor hydroxylation sites to hypoxia and hydroxylase inhibitors. The Journal of Biological Chemistry.

[bib222] Ullah K, Rosendahl AH, Izzi V, Bergmann U, Pihlajaniemi T, Mäki JM, Myllyharju J (2017). Hypoxia-inducible factor prolyl-4-hydroxylase-1 is a convergent point in the reciprocal negative regulation of NF-κB and p53 signaling pathways. Scientific Reports.

[bib223] Villafuerte FC, Corante N (2016). Chronic mountain sickness: Clinical aspects, etiology, management, and treatment. High Altitude Medicine & Biology.

[bib224] Volmer R, van der Ploeg K, Ron D (2013). Membrane lipid saturation activates endoplasmic reticulum unfolded protein response transducers through their transmembrane domains. PNAS.

[bib225] Vozdek R, Long Y, Ma DK (2018). The receptor tyrosine kinase HIR-1 coordinates HIF-independent responses to hypoxia and extracellular matrix injury. Science Signaling.

[bib226] Walport LJ, Hopkinson RJ, Chowdhury R, Schiller R, Ge W, Kawamura A, Schofield CJ (2016). Arginine demethylation is catalysed by a subset of JmjC histone lysine demethylases. Nature Communications.

[bib227] Wang D, Wei Y, Pagliassotti MJ (2006). Saturated fatty acids promote endoplasmic reticulum stress and liver injury in rats with hepatic steatosis. Endocrinology.

[bib228] Ward ME, Toporsian M, Scott JA, Teoh H, Govindaraju V, Quan A, Wener AD, Wang G, Bevan SC, Newton DC, Marsden PA (2005). Hypoxia induces a functionally significant and translationally efficient neuronal NO synthase mRNA variant. Journal of Clinical Investigation.

[bib229] Watschinger K, Keller MA, Golderer G, Hermann M, Maglione M, Sarg B, Lindner HH, Hermetter A, Werner-Felmayer G, Konrat R, Hulo N, Werner ER (2010). Identification of the gene encoding alkylglycerol monooxygenase defines a third class of tetrahydrobiopterin-dependent enzymes. PNAS.

[bib230] Wecksler AT, Jacquot C, van der Donk WA, Holman TR (2009). Mechanistic investigations of human reticulocyte 15- and platelet 12-lipoxygenases with arachidonic acid. Biochemistry.

[bib231] Weits DA, Giuntoli B, Kosmacz M, Parlanti S, Hubberten HM, Riegler H, Hoefgen R, Perata P, van Dongen JT, Licausi F (2014). Plant cysteine oxidases control the oxygen-dependent branch of the N-end-rule pathway. Nature Communications.

[bib232] White MD, Klecker M, Hopkinson RJ, Weits DA, Mueller C, Naumann C, O’Neill R, Wickens J, Yang J, Brooks-Bartlett JC, Garman EF, Grossmann TN, Dissmeyer N, Flashman E (2017). Plant cysteine oxidases are dioxygenases that directly enable arginyl transferase-catalysed arginylation of N-end rule targets. Nature Communications.

[bib233] White MD, Kamps JJAG, East S, Taylor Kearney LJ, Flashman E (2018). The plant cysteine oxidases from *Arabidopsis thaliana* are kinetically tailored to act as oxygen sensors. Journal of Biological Chemistry.

[bib234] Wikström M, Krab K, Sharma V (2018). Oxygen activation and energy conservation by cytochrome c oxidase. Chemical Reviews.

[bib235] Wilks A (2002). Heme oxygenase: evolution, structure, and mechanism. Antioxidants & Redox Signaling.

[bib236] Willam C, Maxwell PH, Nichols L, Lygate C, Tian YM, Bernhardt W, Wiesener M, Ratcliffe PJ, Eckardt K-U, Pugh CW (2006). HIF prolyl hydroxylases in the rat; organ distribution and changes in expression following hypoxia and coronary artery ligation. Journal of Molecular and Cellular Cardiology.

[bib237] Wilson JW, Shakir D, Batie M, Frost M, Rocha S (2020). Oxygen-sensing mechanisms in cells. The FEBS Journal.

[bib238] Windsor JS, Rodway GW (2007). Heights and haematology: the story of haemoglobin at altitude. Postgraduate Medical Journal.

[bib239] Wu X, Zhang Y (2017). TET-mediated active DNA demethylation: mechanism, function and beyond. Nature Reviews. Genetics.

[bib240] Wuren T, Simonson TS, Qin G, Xing J, Huff CD, Witherspoon DJ, Jorde LB, Ge R-L (2014). Shared and unique signals of high-altitude adaptation in geographically distinct Tibetan populations. PLOS ONE.

[bib241] Xie L, Xiao K, Whalen EJ, Forrester MT, Freeman RS, Fong G, Gygi SP, Lefkowitz RJ, Stamler JS (2009). Oxygen-regulated beta(2)-adrenergic receptor hydroxylation by EGLN3 and ubiquitylation by pVHL. Science Signaling.

[bib242] Xu S, Li S, Yang Y, Tan J, Lou H, Jin W, Yang L, Pan X, Wang J, Shen Y, Wu B, Wang H, Jin L (2011). A genome-wide search for signals of high-altitude adaptation in tibetans. Molecular Biology and Evolution.

[bib243] Xu Y, Wang ZA, Green RS, West CM (2012). Role of the Skp1 prolyl-hydroxylation/glycosylation pathway in oxygen dependent submerged development of *Dictyostelium*. BMC Developmental Biology.

[bib244] Yamane K, Toumazou C, Tsukada Y, Erdjument-Bromage H, Tempst P, Wong J, Zhang Y (2006). JHDM2A, a JmjC-containing H3K9 demethylase, facilitates transcription activation by androgen receptor. Cell.

[bib245] Yang J, Jin ZB, Chen J, Huang XF, Li XM, Liang YB, Mao JY, Chen X, Zheng Z, Bakshi A, Zheng DD, Zheng MQ, Wray NR, Visscher PM, Lu F, Qu J (2017). Genetic signatures of high-altitude adaptation in Tibetans. PNAS.

[bib246] Yi X, Liang Y, Huerta-Sanchez E, Jin X, Cuo ZXP, Pool JE, Xu X, Jiang H, Vinckenbosch N, Korneliussen TS, Zheng H, Liu T, He W, Li K, Luo R, Nie X, Wu H, Zhao M, Cao H, Zou J, Shan Y, Li S, Yang Q, Ni P, Tian G, Xu J, Liu X, Jiang T, Wu R, Zhou G, Tang M, Qin J, Wang T, Feng S, Li G, Luosang J, Wang W, Chen F, Wang Y, Zheng X, Li Z, Bianba Z, Yang G, Wang X, Tang S, Gao G, Chen Y, Luo Z, Gusang L, Cao Z, Zhang Q, Ouyang W, Ren X, Liang H, Zheng H, Huang Y, Li J, Bolund L, Kristiansen K, Li Y, Zhang Y, Zhang X, Li R, Li S, Yang H, Nielsen R, Wang J, Wang J (2010). Sequencing of 50 human exomes reveals adaptation to high altitude. Science.

[bib247] Yoshida T, Migita CT (2000). Mechanism of heme degradation by heme oxygenase. Journal of Inorganic Biochemistry.

[bib248] Zaccara S, Ries RJ, Jaffrey SR (2019). Reading, writing and erasing mRNA methylation. Nature Reviews. Molecular Cell Biology.

[bib249] Zhang N, Fu Z, Linke S, Chicher J, Gorman JJ, Visk D, Haddad GG, Poellinger L, Peet DJ, Powell F, Johnson RS (2010). The asparaginyl Hydroxylase factor inhibiting HIF-1α Is an Essential regulator of metabolism. Cell Metabolism.

[bib250] Zheng G, Dahl JA, Niu Y, Fedorcsak P, Huang C-M, Li CJ, Vågbø CB, Shi Y, Wang W-L, Song S-H, Lu Z, Bosmans RPG, Dai Q, Hao Y-J, Yang X, Zhao W-M, Tong W-M, Wang X-J, Bogdan F, Furu K, Fu Y, Jia G, Zhao X, Liu J, Krokan HE, Klungland A, Yang Y-G, He C (2013). ALKBH5 is a mammalian RNA demethylase that impacts RNA metabolism and mouse fertility. Molecular Cell.

[bib251] Zheng X, Zhai B, Koivunen P, Shin SJ, Lu G, Liu J, Geisen C, Chakraborty AA, Moslehi JJ, Smalley DM, Wei X, Chen X, Chen Z, Beres JM, Zhang J, Tsao JL, Brenner MC, Zhang Y, Fan C, DePinho RA, Paik J, Gygi SP, Kaelin WG, Zhang Q (2014). Prolyl hydroxylation by EglN2 destabilizes FOXO3a by blocking its interaction with the USP9x deubiquitinase. Genes & Development.

[bib252] Zhu D, Ran Y (2012). Role of 15-lipoxygenase/15-hydroxyeicosatetraenoic acid in hypoxia-induced pulmonary hypertension. The Journal of Physiological Sciences.

[bib253] Zhuang Q, Feng T, Coleman ML (2015). Modifying the maker: Oxygenases target ribosome biology. Translation.

